# Incidence and prevalence of tuberculosis in incarcerated populations: a systematic review and meta-analysis

**DOI:** 10.1016/S2468-2667(21)00025-6

**Published:** 2021-03-22

**Authors:** Olivia Cords, Leonardo Martinez, Joshua L Warren, Jamieson Michael O’Marr, Katharine S Walter, Ted Cohen, Jimmy Zheng, Albert I Ko, Julio Croda, Jason R Andrews

**Affiliations:** Division of Infectious Diseases and Geographic Medicine, Stanford, CA, USA; Division of Infectious Diseases and Geographic Medicine, Stanford, CA, USA; Department of Epidemiology, School of Public Health, Boston University, Boston, MA, USA; Department of Biostatistics, Yale School of Public Health, New Haven, CT, USA; Division of Infectious Diseases and Geographic Medicine, Stanford, CA, USA; Department of Epidemiology of Microbial Diseases, Yale School of Public Health, New Haven, CT, USA; Division of Infectious Diseases and Geographic Medicine, Stanford, CA, USA; Department of Epidemiology of Microbial Diseases, Yale School of Public Health, New Haven, CT, USA; Stanford University School of Medicine, Stanford, CA, USA; Department of Epidemiology of Microbial Diseases, Yale School of Public Health, New Haven, CT, USA; Oswaldo Cruz Foundation, Salvador, Brazil; Department of Epidemiology of Microbial Diseases, Yale School of Public Health, New Haven, CT, USA; Universidade Federal de Mato Grosso do Sul, Faculdade de Medicina, Campo Grande, Mato Grosso do Sul, Brazil; Fundação Oswaldo Cruz, Campo Grande, Mato Grosso do Sul, Brazil; Division of Infectious Diseases and Geographic Medicine, Stanford, CA, USA

## Abstract

**Background:**

Prisons are recognised as high-risk environments for tuberculosis, but there has been little systematic investigation of the global and regional incidence and prevalence of tuberculosis, and its determinants, in prisons. We did a systematic review and meta-analysis to assess the incidence and prevalence of tuberculosis in incarcerated populations by geographical region.

**Methods:**

In this systematic review and meta-analysis, we searched MEDLINE, Embase, Web of Knowledge, and the LILACS electronic database from Jan 1, 1980, to Nov 15, 2020, for cross-sectional and cohort studies reporting the incidence of *Mycobacterium tuberculosis* infection, incidence of tuberculosis, or prevalence of tuberculosis among incarcerated individuals in all geographical regions. We extracted data from individual studies, and calculated pooled estimates of incidence and prevalence through hierarchical Bayesian meta-regression modelling. We also did subgroup analyses by region. Incidence rate ratios between prisons and the general population were calculated by dividing the incidence of tuberculosis in prisons by WHO estimates of the national population-level incidence.

**Findings:**

We identified 159 relevant studies; 11 investigated the incidence of *M tuberculosis* infection (n=16 318), 51 investigated the incidence of tuberculosis (n=1 858 323), and 106 investigated the prevalence of tuberculosis (n=6 727 513) in incarcerated populations. The overall pooled incidence of *M tuberculosis* infection among prisoners was 15·0 (95% credible interval [CrI] 3·8–41·6) per 100 person-years. The incidence of tuberculosis (per 100 000 person-years) among prisoners was highest in studies from the WHO African (2190 [95% CrI 810–4840] cases) and South-East Asia (1550 [240–5300] cases) regions and in South America (970 [460–1860] cases), and lowest in North America (30 [20–50] cases) and the WHO Eastern Mediterranean region (270 [50–880] cases). The prevalence of tuberculosis was greater than 1000 per 100 000 prisoners in all global regions except for North America and the Western Pacific, and highest in the WHO South-East Asia region (1810 [95% CrI 670–4000] cases per 100 000 prisoners). The incidence rate ratio between prisons and the general population was much higher in South America (26·9; 95% CrI 17·1–40·1) than in other regions, but was nevertheless higher than ten in the WHO African (12·6; 6·2–22·3), Eastern Mediterranean (15·6; 6·5–32·5), and South-East Asia (11·7; 4·1–27·1) regions.

**Interpretation:**

Globally, people in prison are at high risk of contracting *M tuberculosis* infection and developing tuberculosis, with consistent disparities between prisons and the general population across regions. Tuberculosis control programmes should prioritise preventive interventions among incarcerated populations.

**Funding:**

US National Institutes of Health.

## Introduction

Globally, the incidence of tuberculosis was approximately 10 million in 2019, and is decreasing by only 1–2% per year.^1^ This discouragingly low rate of improvement has led to calls for new public health interventions that can improve case detection^2^ by focusing efforts on groups that are at highest risk of developing *Mycobacterium tuberculosis* infection and tuberculosis. WHO has identified several important high-risk groups that have been designated for increased surveillance and preventive therapy interventions, including household contacts of individuals with tuberculosis and children and adults living with HIV.^1,3^ However, to further focus case detection and tuberculosis prevention efforts, a better understanding of the burden of tuberculosis in other high-risk populations is needed.

Globally, in 2018, more than 11 million people were incarcerated.^4^ This number rose by 24% between 2000 and 2018, and increased in nearly all global regions.^4^ In Africa and Asia, regions with the highest global burden of tuberculosis and HIV, the population of incarcerated individuals increased substantially (by 29% in Africa and by 38% in Asia) in these years. Because of high levels of crowding inside prisons, a high prevalence of individual-level risk factors, and lack of access to proper health-care services including diagnosis and treatment, tuberculosis transmission is common and prisoners are generally considered to be at high risk of developing tuberculosis,^5-7^ including drug-resistant forms of the disease.^8,9^ However, much remains unknown about the burden of tuberculosis and the force of *M tuberculosis* infection among prisoners in different regions of the world.^10-12^ Previous systematic reviews focusing on tuberculosis in high-risk populations included small numbers of studies and focused primarily on specific subpopulations (eg, people living with HIV) or a limited range of tuberculosis outcomes.^6,13,14^

We did a systematic review and meta-analysis to investigate the incidence of *M tuberculosis* infection and the incidence and prevalence of tuberculosis among incarcerated populations in all WHO geographical regions. We also assessed whether population characteristics, study design, and regional and setting-specific differences in tuberculosis burden affected the risk of tuberculosis among prisoners.

## Methods

### Search strategy and selection criteria

This systematic review and meta-analysis followed the Preferred Reporting Items for Systematic Reviews and Meta-Analyses (PRISMA) guidelines (appendix pp 35–37).^15^ This study is registered with PROSPERO, CRD42018104463.

We searched MEDLINE, Embase, Web of Knowledge, and the LILACS electronic database from Jan 1, 1980, to Nov 15, 2020, for studies reporting the incidence of *M tuberculosis* infection, incidence of tuberculosis, or prevalence of tuberculosis among incarcerated populations. We chose studies published after 1980 for consistency with a previous meta-analysis on this topic.^6^ The search strategy was developed in consultation with an expert librarian. The search combined terms, keywords, and subject headings based on the following concepts: *M tuberculosis* infection, tuberculosis, and prisons. We used the following search terms, adapted for each database when appropriate: “*Mycobacterium tuberculosis*”, “tuberculosis”, “TB”, “incidence”, “prevalence”, “conversion”, “imprisonment”, “prison”, “inmate”, “transmission”, and “contact*” (the full list of search terms is provided in the appendix pp 6–10). We did not apply any language restrictions.

Two independent reviewers (LM and OC) read and assessed the titles and abstracts of all articles identified by the search strategy. We also included conference abstracts and dissertations if eligible. When reviewing full-text articles, a standardised form containing the inclusion and exclusion criteria was used by each reviewer to record their decisions and comments for study exclusion. The standardised eligibility form was pilot tested on ten studies and was subsequently refined and clarified before the remaining reports were independently reviewed. Any disagreements were resolved through discussion (with LM and OC). Data were extracted from individual studies by four independent reviewers (LM, OC, JZ, and JMO). Data from articles in languages other than English were extracted by LM, OC, and KSW. Data on national tuberculosis incidence were extracted from WHO databases. The mid-point year of the study’s implementation was used for data extraction. When data were extracted, we separated individual studies into distinct cohorts by year of data collection, region, or prison, or all of the above.

Different study designs were used to assess each outcome: cross-sectional surveys for the prevalence of tuberculosis; and cohort studies for the incidence of *M tuberculosis* infection and tuberculosis. A study could contribute data to multiple outcomes if eligible. Studies reporting on only the prevalence of *M tuberculosis* infection, outbreak investigations, case-control studies, and studies published before 1980 were excluded. For studies reporting the incidence of *M tuberculosis* infection or tuberculosis, follow-up had to exceed 6 months.

The WHO definition for prisons is: “institutions that hold people who have been sentenced to a period of imprisonment by the courts for offences against the law.”^16^ We included any form of involuntary detention, including prisons, jails, immigration detention centres, drug detention centres, and other facilities. We use the term prisoner to refer to all incarcerated or detained individuals, as the majority of studies were done in prisons rather than other sites of involuntary detention.

### Data analysis

There were three primary outcomes: incidence of *M tuberculosis* infection, incidence of tuberculosis, and prevalence of tuberculosis. The incidence of *M tuberculosis* infection was defined as the number of prisoners with conversion of a negative tuberculin skin test or interferon-γ release assay to a positive result on repeat testing within a specified time period. Various tuberculin induration cutoffs were used to define conversion. We used each study’s specified definition for conversion. The incidence of tuberculosis was defined as the number of prisoners without tuberculosis at baseline who developed tuberculosis over a specified time period. The prevalence of tuberculosis was defined as the number of cases among prisoners tested for tuberculosis at one specified point in time.

The secondary outcome was the incidence rate ratio (IRR) of tuberculosis between prisoners and the general population. Only studies measuring the incidence of tuberculosis were included in this analysis.

The quality of studies was assessed with a modified version of the Newcastle-Ottawa scale (appendix p 47).^17^ Studies were evaluated on the basis of adequate participant selection (four points), comparability of studies based on design and analysis (one point), and adequate ascertainment of outcomes (three points). This scale awards a maximum of eight points. We defined studies that scored 66·6% or greater as high quality, those that scored 33·3–66·5% as moderate quality, and those that scored less than 33·3% as low quality. Previous systematic reviews were consulted to replicate the methods used.

We combined data from all included studies and nested cohorts in random-effects models. For cohort studies, we calculated follow-up time from the first baseline visit to development of tuberculosis or study completion for each individual cohort and study. For incidence of *M tuberculosis* infection, we included only participants with a negative tuberculin skin test or interferon-γ release assay at baseline. For studies or cohorts, or both, with zero prevalent or incident cases over the follow-up period, we added a fixed value (ie, 0·5).^18^

We evaluated substudy-level predictors of tuberculosis prevalence and incidence using hierarchical Bayesian linear meta-regression models. We opted for a Bayesian framework allowing complete flexibility in modelling our data, including nesting of cohorts within studies as well as the use of multiple predictors of various types. The hierarchical model structure includes two levels of distinct cohorts (level 1: cohort, which includes year of data collection, region, or prison; and level 2: study). The numbers within each study cluster were too small for reliable estimates of the random effects; therefore, we did not present inferences on these parameters. For each subcohort within a study, we calculated a study-specific primary outcome estimate (log odds for prevalence of tuberculosis and log incidence rate for incidence of *M tuberculosis* infection and tuberculosis). We then calculated variance for the estimates and used these combined data to construct statistical models. The models included study-level random intercepts to account for correlation among outcomes caused by the fact that multiple subcohorts were sometimes nested within a single study. Outcomes were then back-transformed after model fitting to report findings on an interpretable scale. Predictors of tuberculosis prevalence and incidence included global region, type of surveillance, study design, facility type, national tuberculosis incidence, designation by WHO as a country with a high tuberculosis burden, and year of study implementation. We investigated whether studies published in different decades had distinct results for our various outcomes through stratification and regression modelling. Because few studies reported incidence of *M tuberculosis* infection, we could not investigate study-level and substudy-level predictors for this outcome. Each individual predictor was investigated in separately fit models for both prevalent and incident tuberculosis outcomes; we also included global region, year of study implementation, and study design.

We also did hierarchical meta-regression to evaluate the burden of tuberculosis among incarcerated people living with HIV or who inject drugs. We restricted this analysis to studies measuring and reporting on these populations.

We also calculated the IRR of tuberculosis among prisoners compared to the general population using the meta-regression model. To do this, we divided study-level tuberculosis incidence in prisons by country-level incidence. Population-level estimates of country-level markers were taken from WHO. For registry-based notification studies, we used case notification rates at the country level; for non-registry-based incidence studies, we used incidence rates at the country level. We were only able to calculate the IRRs from studies published in or after 2000 because WHO does not provide estimates for some countries before this time. We stratified the IRR into subgroups, including global region, national tuberculosis incidence, and national income level. The overall IRR was adjusted for each global region.

For each fitted model, we collected 1 000 000 posterior samples using Markov chain Monte Carlo sampling techniques, after discarding the first 100 000 iterations before convergence. We thinned the collected samples by a factor of 100 to reduce posterior autocorrelation, resulting in 10 000 nearly independent posterior samples. Using these samples, we calculated posterior means (point estimates) and 95% quantile-based credible intervals (95% CrIs) for relevant model parameters to summarise results from the model. Additionally, in the univariable models, we used the output to estimate prevalence and incidence rates for the different levels of the included covariates. Between-study heterogeneity was assessed with the *I*^2^ statistic.^19^ More information about the hierarchical Bayesian meta-regression model is summarised in the appendix (pp 3–5). All statistical analyses were done with R statistical software, with the Rjags package.

### Role of the funding source

The funders of the study had no role in study design, data collection, data analysis, data interpretation, or writing of the report.

## Results

**Figure 1:**
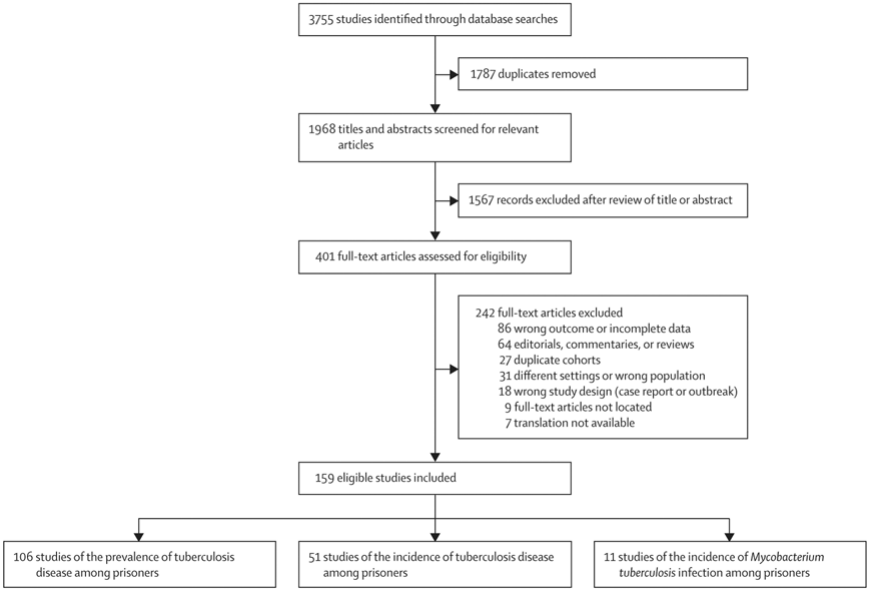
Study selection The total number of eligible studies does not equal the number of eligible studies from the three outcomes because some studies reported more than one outcome. Full-text articles could have been excluded for more than one reason, but only one reason for exclusion was listed for each excluded manuscript.

We found 1968 unique articles from our database searches. 1567 articles were excluded after a review of the title or abstract, leaving 401 articles that were assessed for full-text review, of which 159 studies met the eligibility requirements and were included in the meta-analysis (figure 1; appendix pp 12–31). 11 studies investigated the incidence of *M tuberculosis* infection by use of tuberculin or QuantiFERON (Qiagen) tests, 51 studies investigated the incidence of tuberculosis, and 106 reported the prevalence of tuberculosis (appendix pp 39–45). The number of prisoners within studies reporting the incidence of *M tuberculosis* infection (n=16 318) was lower than for studies reporting the incidence (n=6 727 513) and prevalence of tuberculosis (n=1 858 323).

Data in the 159 published studies were collected from 1976 to 2020. Most studies were published from 2000 to 2020 (77 studies for tuberculosis prevalence and 33 studies for tuberculosis incidence; table 1) and were of moderate or high quality. Most studies came from the WHO Americas region (n=58) followed by the African (n=38) and European regions (n=30; table 1). Seven articles in French, six in Spanish, three in Portuguese, one in Russian, and one in Chinese were included as full-text manuscripts in the meta-analysis. Half the studies provided data on the sex of prisoners and 37 (23%) studies used routine registry-based notification as their method of case detection. Overall, 26 (42%) of 62 studies on the incidence of *M tuberculosis* infection and tuberculosis recruited participants prospectively (table 1). Heterogeneity was substantially high (*I*^2^>98%) in all analyses.

**Figure 2:**
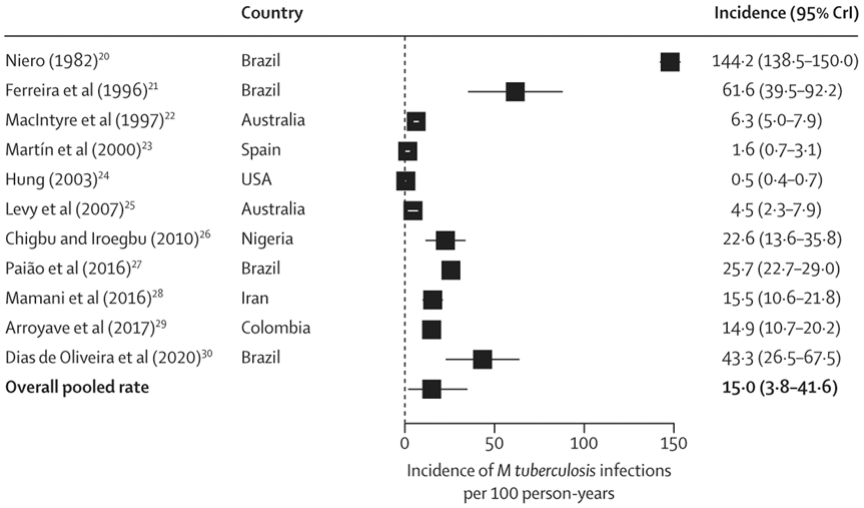
Incidence of *Mycobacterium tuberculosis* infection among incarcerated populations All studies used tuberculin skin tests, except for one, by Dias de Oliveira and colleagues,^30^ which used a QuantiFERON Gold In-Tube test. All pooled estimates were calculated through hierarchical meta-regression modelling. CrI=credible interval.

Of the 11 studies reporting the incidence of *M tuberculosis* infection, seven were from the Americas (four from Brazil, two from the USA, and one from Colombia), and the other four were from Australia, Nigeria, Iran, and Spain. One study used the QuantiFERON Gold In-Tube test (Qiagen), while the rest used the tuberculin skin test. The incidence of *M tuberculosis* infection ranged from one to 144 infections per 100 person-years (figure 2).^20-30^ Infection rates were highest in Brazil and Nigeria (23–144 incident infections per 100 person-years) and lowest in Australia, Spain, and the USA (0–6 infections per 100 person-years), but these low rates were still higher than infection rates among the general population. The overall pooled rate of incident *M tuberculosis* infection among all studies was 15·0 (95% CrI 3·8–41·6) per 100 person-years, based on results from the meta-regression model (figure 2).

Most studies investigating the incidence of tuberculosis were from the WHO Americas region (n=24). Incidence differed markedly across regions. The annual incidence per 100 000 person-years was lowest in North America (30 [95% CrI 20–50] cases) and the WHO Eastern Mediterranean region (270 [50–880] cases; table 2). Incidence was highest in the African (2190 [95% CrI 810–4840] cases) and South-East Asia (1550 [240–4840] cases) regions. Within the Americas, the incidence of tuberculosis was substantially higher in South America (970 [95% CrI 460–1860] cases) than in North America (30 [20–50] cases; table 2). Incidence per 100 000 person-years in countries designated as high-burden countries by WHO was substantially greater (1120 [95% CrI 390–2620] cases) than in those not designated as high-burden countries (160 [90–250] cases). The incidence of tuberculosis in prisons was strongly associated with incidence in the general population (appendix pp 50–51), rising from 40 (95% CrI 20–50) cases per 100 000 person-years in settings with a national tuberculosis incidence of less than ten cases per 100 000 person-years to 2090 (870–4340) cases per 100 000 person-years in settings with a national tuberculosis incidence of 250 or more cases per 100 000 person-years (table 2).

The incidence of tuberculosis per 100 000 person-years was also much higher in prisons (450 [95% CrI 270–690] cases) than in jails (260 [140–460] cases) or detention centres (60 [30–110] cases; table 2).

The largest IRR between prisoners and the general population was in South America (26·9; 95% CrI 17·1–40·1), followed by the Eastern Mediterranean (15·6; 6·5–32·5), African (12·6; 6·2–22·3), South-East Asia (11·7; 4·1–27·1), and European regions (8·7; 3·7–16·8; table 3). The lowest IRR was in North America (4·1; 95% CrI 2·8–6·2).

30 studies investigated the prevalence of tuberculosis in the African region, followed by 28 in the Americas, 18 in the European region, 11 from the Eastern Mediterranean region, ten from the Western Pacific region, and eight from the South-East Asia region. As with incidence, the prevalence of tuberculosis in prisons was strongly associated with the incidence of tuberculosis in the general population (table 2). The prevalence of tuberculosis per 100 000 prisoners rose from 360 (95% CrI 200–600) cases in settings with a national tuberculosis incidence of less than ten cases per 100 000 person-years to 2800 (1730–4220) cases in settings with a national tuberculosis incidence of 250 or more cases per 100 000 person-years.

Among 4845 prisoners living with HIV in 17 tuberculosis prevalence studies, the pooled prevalence was 8210 (95% CrI 3800–15 210) cases per 100 000 prisoners (appendix pp 52–54). The odds of prevalent tuberculosis were higher in prisoners living with HIV (pooled odds ratio 3·6; 95% CrI 2·0–5·9) than in those living without HIV (n=97 129) in these studies. The results from six tuberculosis incidence studies, comprising 3049 prisoners, were statistically unstable because of the small number of studies, and we were unable to reliably calculate a pooled estimate. In five of six studies, people living with HIV had an incidence of more than 3800 cases per 100 000 person-years, although heterogeneity was high (range 395–16 868 cases per 100 000 person-years). There was an insufficient number of studies among people who inject drugs to pool tuberculosis prevalence (four studies; range 0% to 11%) and incidence (two studies; 1240 and 7975 cases per 100 000 person-years) in this population.

## Discussion

In this large systematic review and meta-analysis, we assessed the global incidence of *M tuberculosis* infection and the global prevalence and incidence of tuberculosis in prisons. Tuberculosis incidence rates were consistently much higher in prisons than in the general population, with IRRs ranging from 4·1 in North America to 26·9 in South America, and averaging 10·1 globally. Additionally, overall pooled *M tuberculosis* infection rates were extremely high: 15 new infections per 100 person-years. This work builds on previous reviews of tuberculosis among prisoners^6,11,13^ and provides, to the best of our knowledge, the largest compilation of data on an infectious disease among prisoners to date. Together, these results show that prisons should be prioritised for tuberculosis control efforts in every region of the world.

Despite sustained efforts to decrease the global burden of tuberculosis, incidence is falling at a discouragingly low rate.^1^ Identification of high-risk groups that should be targeted for tuberculosis control interventions is, therefore, a high priority. Despite acknowledgment that incarcerated populations are at high risk of exposure to and development of tuberculosis, prisoners receive scarce attention in major policy documents,^1^ and international reporting of tuberculosis cases among prisoners remains sporadic. As a result, there are few recommendations to implement interventions to increase tuberculosis diagnosis and prevention efforts in this group. WHO guidance recommends active screening among incarcerated populations, but this evidence is considered by WHO to be “conditional” and of “low quality”.^31^

The extent to which tuberculosis risk varies among incarcerated populations in different settings is poorly understood. A previous meta-analysis found high rates of *M tuberculosis* infection and tuberculosis among prisoners, but included few studies from high-burden settings.^6^ We found that in high-burden countries the prevalence and incidence of tuberculosis and incidence of *M tuberculosis* infection was much higher among incarcerated individuals than in the general population. For example, countries with a national tuberculosis incidence of 0–9 cases per 100 000 person-years had a pooled incidence among prisoners of 40 cases per 100 000 person-years, whereas settings with a national incidence of more than 250 cases per 100 000 person-years had a pooled incidence among prisoners of 2090 cases per 100 000 person-years. Additionally, the tuberculosis prevalence of 8210 cases per 100 000 prisoners in incarcerated people living with HIV (more than three times higher than in incarcerated people living without HIV) is striking, and suggests an opportunity for intervention. These disparities have important implications for health policy makers and indicate that targeted tuberculosis control measures in incarcerated populations in high-burden settings could yield substantial benefits and be cost-effective in the long term.

The IRR estimates obtained in this analysis support previous assertions that tuberculosis imparts a large inequitable burden on incarcerated populations. The IRRs we reported were highest in South America but exceeded ten in the WHO African, South-East Asia, and Eastern Mediterranean regions. These IRRs are lower than those reported in a meta-analysis published by Baussano and colleagues in 2010.^6^ There are several potential reasons for this difference. First, Baussano and colleagues estimated a global IRR by calculating the median of IRRs across studies rather than calculating a pooled estimate. Second, we included substantially more studies than this previous meta-analysis and were, therefore, able to show substantial heterogeneity in estimates by global region and background tuberculosis burden. The finding that IRRs were high in low-income settings and in South America, African, and South-East Asia regions suggests that focusing case detection efforts towards prisons might be particularly impactful in countries with a high burden of tuberculosis.

We found a substantially higher annual risk of *M tuberculosis* infection among prisoners compared to previous estimates.^6^ Among 11 studies measuring incidence of *M tuberculosis* infection by use of tuberculin or QuantiFERON tests among prisoners, rates were strikingly high. Among studies done in Iran, Colombia, Nigeria, and Brazil,^20,21,26,27,29,30^ the annual risk of *M tuberculosis* infection was greater than 15%. In four studies in Brazil, the annual risk was greater than 25%, among the highest rates of *M tuberculosis* infection recorded in any population globally.^20,21,27,30^ These rates are orders of magnitude higher than those reported in the general population^32-34^ and in a previous meta-analysis of prisoners.^6^ BCG boosting of tuberculin skin tests in these studies is unlikely as these are conversion studies in which participants must have a baseline negative test. Baussano and colleagues^6^ calculated a median annual *M tuberculosis* infection rate of 2·6 per 100 person-years, which is substantially lower than our estimates. This difference is likely to be multifactorial. Many recent studies have been done in settings with both extensive tuberculosis epidemics and increasing incarceration rates. Additionally, several studies from the analysis by Baussano and colleagues^6^ included guards and prison staff,^35^ who have a lower risk of infection and disease than prisoners.^36^ The elevated *M tuberculosis* infection rates seen in incarcerated populations are concerning and could impede interventions that do not directly prevent transmission in areas with a heavy tuberculosis burden.^5,37^ These results suggest that prisoners are at high risk of developing tuberculosis in every global region and should be prioritised for tuberculosis screening. Preventing sources of tuberculosis transmission through mass screening, case isolation, or decreasing crowding might be essential to reduce subsequent progression to tuberculosis. Reducing transmission in prisons could also prevent spillover of tuberculosis from prisons into the general population.^7,9,38^

There are several limitations to this analysis. First, we found marked heterogeneity between studies. Although characteristics such as global region, income status, and background tuberculosis burden predicted tuberculosis rates among prisoners, variability from study-level estimates could have come from unmeasured characteristics. Second, there were substantial study-level differences in how data were collected and reported, screening approaches, and definitions of tuberculosis. We classified and grouped studies according to their data collection procedures; however, there were considerable residual differences in case detection procedures that could have contributed to variability in outcome estimates. Third, there was a lack of detailed reporting of individual-level and prison-level characteristics, such as prison crowding or prevalence of medical comorbidities that influence tuberculosis risk, restricting our ability to identify specific traits driving the increased tuberculosis burden. Fourth, publication bias is possible in even the most thorough systematic review. Although we searched multiple electronic databases as well as the grey literature, we could have missed articles that were difficult to find. Finally, although drug resistance has been reported to be an important problem in prisons,^8,9^ few studies systematically measured the prevalence or incidence of drug-resistant tuberculosis, preventing us from being able to evaluate the extent of this problem.

In conclusion, we found an extremely high incidence of *M tuberculosis* infection as well as an extremely high prevalence and incidence of tuberculosis among prisoners worldwide. Although the incidence of tuberculosis was higher in prisoners than in the general population in all settings, this disparity was greatest in South America and considerably high in the WHO African, Eastern Mediterranean, and South-East Asia regions. International guidelines and national tuberculosis programmes focus on case detection and preventive interventions for certain high-risk populations (eg, people living with HIV and household contacts of individuals with confirmed tuberculosis), but there has been comparatively less emphasis on incarcerated populations.^11,31^ The extraordinarily high burden of tuberculosis in prisons identified consistently in studies worldwide indicates that incarcerated individuals should be prioritised in international recommendations and tuberculosis-reporting metrics. National tuberculosis control programmes should develop and invest in robust active case finding and preventive interventions to address the crisis of tuberculosis in prisons and other detention facilities.

## Supplementary Material

1

## Figures and Tables

**Table 1:** Studies included in the systematic review

	Studies of incidence of *Mycobacterium tuberculosis* infection (n=11)*	Studies of incidence of tuberculosis (n=51)*	Studies of prevalence of tuberculosis (n=106)*
**Year of study implementation**†			
1970–79	1 (9%)	3 (6%)	2 (2%)
1980–89	0 (0)	3 (6%)	2 (2%)
1990–99	4 (36%)	22 (43%)	24 (23%)
2000–09	2 (18%)	15 (29%)	40 (38%)
2010–20	4 (36%)	18 (35%)	37 (35%)
Prospective study design‡	9 (82%)	18 (35%)	..
Tested for HIV§	7 (64%)	16 (31%)	38 (36%)
Registry-based notification	1 (9%)	33 (65%)	3 (3%)
Data on sex status	9 (82%)	13 (25%)	64 (60%)
WHO high burden	6 (55%)	14 (27%)	54 (51%)
**National incidence per 100 000 person-years**			
0–9	3 (27%)	11 (22%)	23 (22%)
10–49	5 (45%)	20 (39%)	25 (24%)
50–149	2 (18%)	9 (18%)	14 (13%)
150–249	1 (9%)	5 (10%)	18 (17%)
≥250	0	6 (12%)	27 (26%)
**Global region**¶			
African	1 (9%)	7 (14%)	30 (28%)
North America	2 (18%)	12 (23%)	12 (11%)
South America	5 (45%)	12 (23%)	17 (16%)
Eastern Mediterranean	1 (9%)	2 (4%)	11 (10%)
European	1 (9%)	11 (22%)	18 (17%)
South-East Asia	0	3 (6%)	8 (8%)
Western Pacific	1 (9%)	3 (6%)	10 (9%)
**Facility type**			
Prison	10 (91%)	39 (77%)	73 (69%)
Combination of detention centres	1 (9%)	7 (14%)	10 (9%)
Jail	0	4 (8%)	15 (14%)
Detention centre	0	1 (2%)	1 (1%)
Immigration detention	0	0	5 (5%)
Juvenile detention centre	0	0	1 (1%)
Psychiatric facility	0	0	1 (1%)

* Some cohorts contributed to more than one outcome (incidence of *Mycobacterium tuberculosis* infection, incidence of tuberculosis, and prevalence of tuberculosis) so percentages for these categories do not add up to 100%.

† One study could be included in more than one category for this variable if the study was implemented during multiple timepoints; for example, if the study was done from 1998 to 2002, it would be included in both the 1990–99 and 2000–09 categories. Therefore, the total number of studies for each outcome might not be congruent with the total number listed for this characteristic.

‡ Includes only incident cohorts.

§ Some or all participants were tested for HIV.

¶ WHO classifies the Americas as one region; because of substantial differences in tuberculosis burden among incarcerated populations in North America and South America, we separated out this region.

**Table 2:** Pooled prevalence and incidence of tuberculosis among incarcerated populations, stratified by key subgroups*

	Prevalence studies		Incidence studies	
	Number of studies	Prevalence per 100 000 prisoners (95% CrI)*	Number of studies	Incidence per 100 000 person-years (95% CrI)*
**Global region**†				
North America	12	320 (130–650)	12	30 (20–50)
South America	16	1680 (830–2970)	12	970 (460–1860)
European	18	1000 (510–1770)	11	610 (310–1100)
African	30	1610 (980–2500)	7	2190 (810–4840)
South-East Asia	8	1810 (670–4000)	3	1550 (240–5300)
Western Pacific	10	720 (270–1600)	3	390 (80–1130)
Eastern Mediterranean	11	1160 (480–2370)	3	270 (50–880)
**WHO high-burden country**‡				
No	52	860 (630–1440)	37	160 (90–250)
Yes	53	1470 (1000–2090)	14	1120 (390–2620)
**National incidence per 100 000 person-years**				
0–9	23	360 (200–600)	11	40 (20–50)
10–49	24	1320 (800–2040)	20	480 (300–710)
50–149	14	980 (510–1720)	9	930 (470–1640)
150–249	18	920 (500–1540)	5	1530 (700–2930)
≥250	27	2800 (1730–4220)	6	2090 (870–4340)
**Type of surveillance**				
Passive surveillance	6	1370 (640–2590)	12	520 (210–1060)
Active surveillance	97	1120 (830–1470)	31	440 (240–720)
Not specified	..	..	9	100 (50–170)
**Study design**				
Study-based	89	1190 (890–1540)	18	1430 (580–2950)
Registry-based notification	3	290 (20–1210)	30	110 (70–200)
**Facility type**				
Prison	72	1370 (980–1850)	47	450 (270–690)
Combination of detention centres	10	1350 (450–3120)	30	60 (30–110)
Detention centre	1	480 (120–870)	..	..
Jail	15	910 (400–2090)	4	260 (140–460)
Study years of data collection§				
1970–89	4	1470 (450–3590)	5	100 (20–310)
1990–99	24	1110 (610–1840)	22	330 (180–560)
2000–09	40	1070 (660–1620)	15	270 (150–440)
2010–20	36	1230 (760–1840)	18	280 (150–450)

CrI=credible interval.

* All pooled estimates were calculated through hierarchical meta-regression modelling.

† WHO classifies the Americas as one region; because of substantial differences in tuberculosis burden among incarcerated populations in North America and South America, we separated out this region.

‡ WHO classifies 30 countries as “high-burden countries”, which includes the top 20 countries in terms of absolute numbers of cases plus the additional ten countries with the most severe burden in terms of case rates per capita that do not already appear in the top 20. Each country on this list must meet a minimum threshold in terms of absolute numbers of cases (10 000 per year for tuberculosis).

§ Because of the low number of studies in the 1970s and 1980s, we grouped these two decades into one group for this outcome.

**Table 3:** Incidence rate ratios between prisoners and the general population, stratified by global region, national incidence, and country income status

	Number of studies	Number of cohorts	Incidence rate ratio (95% CrI)
Overall*	47	270	10·1 (7·6–13·0)
Global region†			
North America	7	98	4·1 (2·8–6·2)
South America	12	34	26·9 (17·1–40·1)
European	6	29	8·7 (3·7–16·8)
African	6	22	12·6 (6·2–22·3)
South-East Asia	3	44	11·7 (4·1–27·1)
Western Pacific	3	29	6·8 (2·9–13·2)
Eastern Mediterranean	3	24	15·6 (6·5–32·5)
National incidence per 100 000 person-years			
0–9	15	97	5·0 (3·3–7·7)
10–49	19	82	17·5 (11·9–26·6)
50–149	6	16	12·1 (6·4–21·4)
150–249	7	53	11·0 (6·1–18·3)
≥250	4	22	8·9 (3·9–17·9)
National income			
Low	6	13	16·4 (8·5–28·9)
Lower middle	14	86	10·6 (6·4–16·5)
Upper middle	17	46	18·2 (11·7–26·3)
High	17	125	4·9 (3·3–7·3)

CrI=credible interval. Only studies measuring tuberculosis incidence were included. To compare incidence rates from two different groups on the same scale, the incidence rate ratio is the incidence in prisons divided by the incidence in the country. Population-level estimates were taken from WHO estimates of country-level markers. For case-notification studies, we used case notification rates at the country level; for non-case notification incidence studies, we used incidence rates at the country level. We were only able to calculate the incidence rate ratio from studies implemented in or after 2000 because WHO does not provide estimates for some countries before this time. Therefore, studies published before this period were not included and the number of studies (by global region) is not congruent with the number of studies shown in tables 1 and 2.

* The overall incidence rate ratio was adjusted for global region considering the wide heterogeneity seen in this variable.

† WHO classifies the Americas as one region; because of substantial differences in tuberculosis burden among incarcerated populations in North and South America, we separated out this region.

## Research in context

### Evidence before this study

Three previous systematic reviews have shown that the incidence of tuberculosis and other infectious diseases is high among incarcerated individuals. However, these studies did not evaluate the prevalence of tuberculosis in incarcerated populations. Additionally, these systematic reviews included small numbers of studies and focused primarily on specific subpopulations (eg, people living with HIV) or a limited range of tuberculosis outcomes. A comparison of tuberculosis-related outcomes among prisoners in high-burden versus lower-burden settings was not possible in these studies, and therefore our understanding of the transmission dynamics of tuberculosis and its prevalence in prisons and other detention facilities from high-burden settings is not well elucidated. WHO guidance recommends active screening among incarcerated populations; however, the evidence of the benefits of such screening is considered by WHO to be “conditional” and of “low quality”.

### Added value of this study

In this systematic review and meta-analysis, we identified 159 studies investigating the prevalence or incidence of tuberculosis or incidence of *Mycobacterium tuberculosis* infection in incarcerated populations. This study provides, to the best of our knowledge, the largest compilation of data on an infectious disease among prisoners. We found an overall pooled incidence rate of *M tuberculosis* infection of 15 per 100 person-years, and more than 20 new infections per 100 person-years in five of 11 studies. The incidence of tuberculosis (per 100 000 person-years) among prisoners was highest in studies from Africa, South-East Asia, and South America, and lowest in North America. The prevalence of tuberculosis was greater than 1000 per 100 000 prisoners in all global regions except for North America and the Western Pacific. In the WHO South-East Asia region the prevalence of tuberculosis was 1810 cases per 100 000 prisoners. We also found that the incidence rate ratio between prisons and the general population was much higher in South America than in other WHO regions but was higher than ten in the African, Eastern Mediterranean, and South-East Asia regions. These results build on previous analyses and provide a greater scope globally of the burden of tuberculosis among incarcerated populations.

### Implications of all the available evidence

Globally, prisoners are at high risk of contracting *M tuberculosis* infection and developing tuberculosis, with consistent disparities observed between prisons and the general population across all geographical regions. Our results suggest that tuberculosis control programmes should prioritise interventions among incarcerated populations and that people who are in detention should be considered a high-risk population in global guidelines on tuberculosis screening.
