# Supplementary material for: Incidence and prevalence of tuberculosis in incarcerated populations: a systematic review and meta-analysis
Source: Lancet Public Health. Author manuscript; Available in PMC 2021 Jun 1. (PMC8168455; doi:10.1016/S2468-2667(21)00025-6)
Supplement: 1 [file NIHMS1698678-supplement-1.pdf]

THE LANCET
Public Health

Supplementary appendix

This appendix formed part of the original submission and has been peer reviewed. We post it as supplied by the authors.

Supplement to: Cords O, Martinez L, Warren JL, et al. Incidence and prevalence of tuberculosis in incarcerated populations: a systematic review and meta-analysis. *Lancet Public Health* 2021; published online March 22. http://dx.doi.org/10.1016/S2468-2667(21)00025-6.

Supplementary Appendix.

Supplement to: Incidence and Prevalence of Tuberculosis in Incarcerated Populations:
A Systematic Review and Meta-analysis

Table of Contents.

1. Additional Methodological Information.

2. Search Strategy.

3. Author Contributions.

4. References for All Individual Studies

5. Supplementary Table 1. Multivariable Bayesian meta-regression analysis of prevalent and incident tuberculosis among prisoners from studies included in the systematic review.

6. Supplementary Table 2. Distribution of articles by languages.

7. Supplementary Table 3. Checklist of items to include when reporting a systematic review or meta-analysis

8. Supplementary Table 4. Study characteristics for studies included in the incidence of tuberculosis infection outcome

9. Supplementary Table 5. Study characteristics for studies included in the incidence of tuberculosis outcome

10. Supplementary Table 6. Study characteristics for studies included in the prevalence of tuberculosis outcome

11. Supplementary Table 5. Study quality of included studies.

12. Study Quality Assessment

13. Supplementary Figure 1. Availability of study-level data from meta-analysis on tuberculosis among prisons.

14. Supplementary Figure 2. Tuberculosis incidence per 100 thousand person-years among prisoners in different detention facilities in countries with cohort-level data.

15. Supplementary Figure 3. The relationship between with study-specific tuberculosis incidence in prisons and tuberculosis incidence in the general population

16. Supplementary Table 6. Prevalence and increased odds of tuberculosis among persons living with HIV deprived of liberty.

17. Supplementary Table 7. Incidence and increased risk of tuberculosis among persons living with HIV deprived of liberty.

1. Additional Methodological Information.

Statistical Method for Tuberculosis Prevalence and Incidence:

$$\hat{\theta}_{ij} \sim \mathrm{N}\left(\theta_{ij}, \hat{\tau}_{ij}{}^{2}\right);\ i = 1, \dots, n;\ j = 1, \dots, m_i$$

$$\theta_{ij} \sim \mathrm{N}\left(\mathbf{x}_{ij}^{\mathrm{T}}\boldsymbol{\beta} + \eta_i 1(m_i > 1), \sigma_{1(m_i>1)}^{2}\right)$$

$$\eta_i \sim \mathrm{N}\left(0, \sigma_{\eta}^{2}\right), i \in \{k = 1, \dots, n{:}\, m_k > 1\}$$

Priors:

$$\beta_k \sim \mathrm{N}(0, 100^2), k = 0, \dots, p$$

$$\sigma_0, \sigma_1, \sigma_\eta \sim \mathrm{Uniform}(0,1000)$$

- $\hat{\theta}_{ij}$: Estimated outcome from cohort *j* within study *i*
- n: Total number of unique studies
- m_i: Total number of cohorts within study *i*
- $\hat{\tau}_{ij}$: Standard error of the outcome
- θ_{ij}: True but unobserved outcome
- $\mathbf{x}_{ij}$: Vector of covariates
- $\boldsymbol{\beta}$: Vector of regression parameters describing the association between the covariates and the outcome
- η_i: Random effect specific to study *i*
- $1(m_i > 1)$: Indicator function that is equal to one if study *i* includes more than one cohort ($m_i > 1$) and is equal to zero if study *i* only includes a single cohort ($m_i = 1$)
- σ_0: Standard deviation parameter for studies that only included a single cohort
- σ_1: Standard deviation parameter for studies that included more than one cohort
- σ_η: Standard deviation parameter that describes within study variation/correlation

Statistical Method for Incident Tuberculosis Infection:

$$\hat{\theta}_i \sim \mathrm{N}\left(\theta_i, \hat{\tau}_i{}^{2}\right);\ i = 1, \dots, n$$

$$\theta_i \sim \mathrm{N}(\beta_0, \sigma^2)$$

Priors:

$$\beta_0 \sim \mathrm{N}(0,100^2)$$

$$\sigma \sim \mathrm{Uniform}(0,1000)$$

Hierarchical Bayesian Meta-Regression Model:

$$\hat{\theta}_{ij}|\theta_{ij} \sim \mathrm{N}(\theta_{ij}, \hat{\sigma}_{ij}^2)$$

$$\theta_{ij} = \mathbf{x}_{ij}^{\mathrm{T}}\boldsymbol{\beta} + \eta_j + \epsilon_{ij} \text{ for studies with multiple cohorts}$$

$$\theta_{ij} = \mathbf{x}_{ij}^{\mathrm{T}}\boldsymbol{\beta} + \kappa_{ij} \text{ for studies with a single cohort}$$

- $\hat{\theta}_{ij}$: Outcome corresponding to cohort *i* nested within study *j*
  - Log odds ratios for prevalence analysis
  - Log relative risks for incidence analysis
- $\hat{\sigma}_{ij}$: Standard error of the outcome
- θ_{ij}: True outcome; unobserved, only measured with error
- $\mathbf{x}_{ij}$: Vector of covariates that describe variability in the true outcome
- $\boldsymbol{\beta}$: Regression parameters of interest
- $\eta_j|\sigma_\eta^2 \sim \mathrm{N}(0, \sigma_\eta^2)$: Study-specific random effect; accounts for correlation in the outcome among cohorts from the same study
- $\epsilon_{ij}|\sigma_\epsilon^2 \sim \mathrm{N}(0, \sigma_\epsilon^2)$: Error term for outcomes from studies with multiple cohorts
- $\kappa_{ij}|\sigma_\kappa^2 \sim \mathrm{N}(0, \sigma_\kappa^2)$: Error term for outcomes from studies with a single cohort

Prior Distributions:

- $\beta_j \sim \mathrm{N}(0, 100^2)$
- $\sigma_\eta, \sigma_\epsilon, \sigma_\kappa \sim \mathrm{Uniform}(0, 1000)$

2. Search Strategy.

PubMed, Web of Science, EMBASE, Biosis, LILACS, INDMED

Pubmed – 1,094

Search completed August 6, 2020

(“tuberculosis"[MeSH Terms] OR "tuberculosis, multidrug-resistant"[MeSH Terms] OR “Mycobacterium tuberculosis”[MeSH] OR “Mycobacterium tuberculosis”[Text Word] OR “MDR -TB”[tw] OR “XDR - TB”[Text Word] OR “Mtb”[tw] OR (extensively[All Fields] AND ("drug resistance"[MeSH Terms] OR drug-resistant[Text Word])))

AND

(detention[All Fields] OR jail[All Fields] OR gaol[All Fields] OR "correctional facility"[All Fields] OR "correctional facilities"[All Fields] OR incarcerat*[All Fields] OR imprisonment[All Fields] OR confinement[All Fields] OR inmates[All Fields] OR "prisons"[MeSH Terms] OR "prison*" [All Fields] OR Penitentiar*[tw] OR imprison*[tw] OR penal OR Criminals [MesH] OR "Concentration Camps" OR inmate*[tw] OR "Correctional setting"[tw] OR "Correctional settings"[tw] OR detain*[tw] OR detention*[tw] OR "Correctional Centre" [tw] OR "Correctional Centres"[tw] OR “compulsory drug detention” [tw] OR “compulsory drug treatment” OR “correction center” [tw] OR “laojiaosuo”[tw] OR “long-term detention” [tw] OR labor camp* [tw])

AND

("1980/01/01"[PDat] : "3000/12/31"[PDat])

Embase – 1,151

Search completed August 6, 2020

('tuberculosis'/de OR 'multidrug resistance' OR 'mycobacterium tuberculosis'/exp OR 'mdr -tb' OR 'xdr -tb' OR 'mtb' OR 'tb'/de)

AND

('detention'/de OR jail OR gaol OR 'correctional facility' OR prison OR incarcerat* OR 'imprison*' OR detain* OR 'correction center' OR 'long-term detention' OR 'correctional settings' OR inmate*)

AND

(1980:py OR 1981:py OR 1982:py OR 1983:py OR 1984:py OR 1985:py OR 1986:py OR 1987:py OR 1988:py OR 1989:py OR 1990:py OR 1991:py OR 1992:py OR 1993:py OR 1994:py OR 1995:py OR 1996:py

OR 1997:py OR 1998:py OR 1999:py OR 2000:py OR 2001:py OR 2002:py OR 2003:py OR 2004:py OR 2005:py OR 2006:py OR 2007:py OR 2008:py OR 2009:py OR 2010:py OR 2011:py OR 2012:py OR 2013:py OR 2014:py OR 2015:py OR 2016:py OR 2017:py OR 2018:py OR 2019:py OR 2020:py)

<u>Web of Science – 867</u>

Search completed August 6, 2020

Search was restricted to articles after January 1, 1980 until the date of the search.

((TS=tuberculosis) OR TS=('mycobacterium tuberculosis') OR TS=(TB) OR TS=("MDR-TB") OR TS=("XDR-TB"))

AND

(TS=(detention) OR TS=(jail) OR TS=(gaol) OR TS=("correctional facility") OR TS=("correctional facilities") OR TS=(incarcerat*) OR TS=(imprisonment) OR TS=(confinement) OR TS=(inmates) OR TS=("prisons") OR TS=(imprison*) OR TS=(penal) OR TS=("Correctional Centres") OR TS=("Correctional Centre") OR TOPIC: (detain*) OR TS=(detention*) OR TS=("correction center") OR TS=("Concentration Camps") OR TS=(inmate*) OR TS=("long-term detention"))

<u>BIOSIS – 463</u>

Search completed August 6, 2020

Search was restricted to articles after January 1, 1980 until the date of the search.

((TS=tuberculosis) OR TS=('mycobacterium tuberculosis') OR TS=(TB) OR TS=("MDR-TB") OR TS=("XDR-TB"))

AND

(TS=(detention) OR TS=(jail) OR TS=(gaol) OR TS=("correctional facility") OR TS=("correctional facilities") OR TS=(incarcerat*) OR TS=(imprisonment) OR TS=(confinement) OR TS=(inmates) OR TS=("prisons") OR TS=(imprison*) OR TS=(penal) OR TS=("Correctional Centres") OR TS=("Correctional Centre") OR TOPIC: (detain*) OR TS=(detention*) OR TS=("correction center") OR TS=("Concentration Camps") OR TS=(inmate*) OR TS=("long-term detention"))

<u>LILACS – 137</u>

Search completed August 6, 2020

Search was restricted to articles after January 1, 1980 until the date of the search.

(tuberculosis [Words])

AND

(prison [Words] or jail [Words])

<u>INDMED – 3</u>

Search completed August 6, 2020

Search was restricted to articles after January 1, 1980 until the date of the search.

(tuberculosis [Any Field])

AND

(prison [Any Field] or jail [Any Field])

Total from Search: 3,715

Total from Search after exclusion of duplicates: 1,928

An additional search was done after from August 2020 to November 15, 2020. The results from this search can be seen below.

PubMed:

("tuberculosis"[MeSH Terms] OR "tuberculosis, multidrug-resistant"[MeSH Terms] OR "Mycobacterium tuberculosis"[MeSH] OR "Mycobacterium tuberculosis"[Text Word] OR "MDR -TB"[tw] OR "XDR - TB"[Text Word] OR "Mtb"[tw] OR (extensively[All Fields] AND ("drug resistance"[MeSH Terms] OR drug-resistant[Text Word]))) AND (detention[All Fields] OR jail[All Fields] OR gaol[All Fields] OR "correctional facility"[All Fields] OR "correctional facilities"[All Fields] OR incarcerat*[All Fields] OR imprisonment[All Fields] OR confinement[All Fields] OR inmates[All Fields] OR "prisons"[MeSH Terms] OR "prison*" [All Fields] OR Penitentiar*[tw] OR imprison*[tw] OR penal OR Criminals [MesH] OR "Concentration Camps" OR inmate*[tw] OR "Correctional setting"[tw] OR "Correctional settings"[tw] OR detain*[tw] OR detention*[tw] OR "Correctional Centre" [tw] OR "Correctional Centres"[tw] OR "compulsory drug detention" [tw] OR "compulsory drug treatment" OR "correction center" [tw] OR "laojiaosuo"[tw] OR "long-term detention" [tw] OR labor camp* [tw]) AND ("2020/08/01"[PDat] : "3000/12/31"[PDat])

7 articles

Embase:

('tuberculosis'/de OR 'multidrug resistance' OR 'mycobacterium tuberculosis'/exp OR 'mdr -tb' OR 'xdr -tb' OR 'mtb' OR 'tb'/de) AND ('detention'/de OR jail OR gaol OR 'correctional facility' OR prison OR incarcerat* OR 'imprison*' OR detain* OR 'correction center' OR 'long-term detention' OR 'correctional settings' OR inmate*) AND (2020:py) AND ([1-8-2020]/sd)

25 articles

Web of Science:

((TS=tuberculosis) OR TS=('mycobacterium tuberculosis') OR TS=(TB) OR TS=("MDR-TB") OR TS=("XDR-TB"))

AND

(TS=(detention) OR TS=(jail) OR TS=(gaol) OR TS=("correctional facility") OR TS=("correctional facilities") OR TS=(incarcerat*) OR TS=(imprisonment) OR TS=(confinement) OR TS=(inmates) OR TS=("prisons") OR TS=(imprison*) OR TS=(penal) OR TS=("Correctional Centres") OR TS=("Correctional Centre") OR TS=(detain*) OR TS=(detention*) OR TS=(“correction center”) OR TS=("Concentration Camps") OR TS=(inmate*) OR TS=(“long-term detention”))

Refined by: PUBLICATION YEARS: (2020)

8 articles

Biosis:

((TS=tuberculosis) OR TS=('mycobacterium tuberculosis') OR TS=(TB) OR TS=("MDR-TB") OR TS=("XDR-TB"))

AND

(TS=(detention) OR TS=(jail) OR TS=(gaol) OR TS=("correctional facility") OR TS=("correctional facilities") OR TS=(incarcerat*) OR TS=(imprisonment) OR TS=(confinement) OR TS=(inmates) OR TS=("prisons") OR TS=(imprison*) OR TS=(penal) OR TS=("Correctional Centres") OR TS=("Correctional Centre") OR TS=(detain*) OR TS=(detention*) OR TS=(“correction center”) OR TS=("Concentration Camps") OR TS=(inmate*) OR TS=(“long-term detention”))

Refined by: PUBLICATION YEARS: (2020)

0 articles

For this additional search, Biosis and Web of Science we manually reviewed the articles from August 2020 onwards (as there was no simple way to classify the search by month). Therefore, we have added 30 articles to the flowchart accordingly.

3. Author Contributions.

Olivia Cords, Leonardo Martinez, Joshua Warren, Jamieson Michael O'Marr, Katharine S. Walter, Ted Cohen, Jimmy Zheng, Albert I. Ko, Julio Croda, Jason R. Andrews

LM, OC, and JRA conceived the study. LM and OC did the systematic search. LM and OC screened and identified eligible studies. LM and OC made final decisions about inclusion of articles. LM, OC, JMO, and JZ extracted data from eligible studies. LM, OC, and KSW extracted data from studies in languages other than English. LM, OC, JW, TC, and JRA designed the analyses. OC and LM received and checked the data. OC, LM, JW, and JRA did the analyses and had full access to all materials and results. LM, OC, and JRA created the figures. LM wrote the first draft of the manuscript. OC, JRA, and JW helped revise the drafted version before and after circulation to collaborators. All authors provided input on interpretation of results. All authors edited subsequent drafts of the manuscript. All authors read and approved the final version of the manuscript. The corresponding author had access to all the data in the study and had final responsibility for the decision to submit for publication. LM affirms that the manuscript is an honest, accurate, and transparent account of the study being reported, and that no important aspects of the study have been omitted. All discrepancies have been explained.

4. References for All Individual Studies

References for the Outcome of Incident Tuberculosis

Aguilera, X.P., González, C., Nájera-De Ferrari, M., Hirmas, M., Delgado, I., Olea, A., Lezaeta, L., Montana, A., Gonzalez, P., Hormazabal, J.C. and Fernández, J., 2016. Tuberculosis in prisoners and their contacts in Chile: estimating incidence and latent infection. The International Journal of Tuberculosis and Lung Disease, 20(1), pp.63-70.

Alavi, S.M., Bakhtiarinia, P., Eghtesad, M., Albaji, A. and Salmanzadeh, S., 2014. A comparative study on the prevalence and risk factors of tuberculosis among the prisoners in khuzestan, South-west iran. Jundishapur Journal of Microbiology, 7(12).

Auregan, G., Rakotomanana, F., Ratsitorahina, M., Rakotoniaina, N., Rabemananjara, O., Raharimanana, R. and Boisier, P., 1995. Tuberculosis in the prison milieu at Antananarivo from 1990 to 1993. Archives de L'institut Pasteur de Madagascar, 62(1), pp.18-23.

Ayala, G., Garay, J., Aragon, M., Decroo, T. and Zachariah, R., 2016. Trends in tuberculosis notification and treatment outcomes in prisons: a country-wide assessment in El Salvador from 2009–2014. Revista Panamericana de Salud Pública, 39, pp.38-43.

Baillargeon, J., Kelley, M., Lichtenstein, M.J., Jenson, H.B. and Linthicum, L., 2002. Management of tuberculosis in the Texas prison system. Journal of Correctional Health Care, 9(1), pp.77-93.

Braun, M.M., Truman, B.I., Maguire, B., DiFerdinando, G.T., Wormser, G., Broaddus, R. and Morse, D.L., 1989. Increasing incidence of tuberculosis in a prison inmate population: association with HIV infection. Jama, 261(3), pp.393-397.

Brock, N.N., Reeves, M., LaMarre, M. and Devoe, B., 1998. Tuberculosis case detection in a state prison system. Public Health Reports, 113(4), p.359.

Bubochkin B. Epidemiological situation in relation to tuberculosis in penalty institutions. Problemy Tuberkulosa 1995; 3: 7-9.

Castañeda-Hernández, D.M., Martínez-Ramírez, J.E., Bolivar-Mejía, A. and Rodríguez-Morales, A.J., 2013. Differences in TB incidence between prison and general populations, Pereira, Colombia, 2010-2011. Tuberculosis (Edinburgh, Scotland), 93(3), pp.275-276.

Chaves, F., Dronda, F., González, A.L., Fernández, F.G. and Catalan, S., 1993. Tuberculosis in a prison population: a study of 138 cases. Medicina clinica, 101(14), pp.525-529.

Chaves, F., Dronda, F., Cave, M.D., Alonso-Sanz, M., Gonzalez-Lopez, A., Eisenach, K.D., Ortega, A., Lopez-Cubero, L., Fernandez-Martin, I., Catalan, S. and Bates, J.H., 1997. A longitudinal study of transmission of tuberculosis in a large prison population. American journal of respiratory and critical care medicine, 155(2), pp.719-725.

Chigbu, L.N. and Iroegbu, C.U., 2010. Incidence and spread of Mycobacterium tuberculosis-associated infection among Aba Federal prison inmates in Nigeria. Journal of health, population, and nutrition, 28(4), p.327.

Costa-Junior, A.O., da Silva Júnior, J.L.R., da Costa, A.C., Kipnis, A., Rabahi, M.F. and Junqueira-Kipnis, A.P., 2016. Tuberculose latente em indivíduos de uma unidade prisional do centro oeste do Brasil. Revista de Patologia Tropical/Journal of Tropical Pathology, 45(1), pp.12-22.

de Oliveira, H.B. and Cardoso, J.C., 2004. Tuberculosis among city jail inmates in Campinas, Sao Paulo, Brazil. Pan American Journal of Public Health, 15(3), pp.194-199

Degner, N.R., Joshua, A., Padilla, R., Vo, H.H. and Vilke, G.M., 2016. Comparison of digital chest radiography to purified protein derivative for screening of tuberculosis in newly admitted inmates. Journal of Correctional Health Care, 22(4), pp.322-330.

Dhuria, M., Sharma, N., Chopra, K.K. and Chandra, S., 2016. Universal access to DOTS in Delhi Prisons: Where do we stand?. Indian Journal of Tuberculosis, 63(1), pp.39-43.

Fernandez de la Hoz, K., Inigo, J., Fernandez-Martín J, I., Arce, A., Alonso-Sanz, M., Gomez-Pintado, P., Palenque, E. and Chaves, F., 2001. The influence of HIV infection and imprisonment on dissemination of Mycobacterium tuberculosis in a large Spanish city. The International Journal of Tuberculosis and Lung Disease, 5(8), pp.696-702.

Ferreira, M.M., Ferrazoli, L., Palaci, M., Salles, P.S., Medeiros, L.A., Novoa, P., Kiefer, C.R., Schechtmann, M., Kritski, A.L., Johnson, W.D. and Riley, L.W., 1996. Tuberculosis and HIV infection among female inmates in Sao Paulo, Brazil: a prospective cohort study. JAIDS Journal of Acquired Immune Deficiency Syndromes, 13(2), pp.177-183.

Fountain, J.F., 1997. Tuberculosis in Shelby County and Tennessee correctional facilities. Tennessee medicine: journal of the Tennessee Medical Association, 90(4), pp.138-140.

Gurbanova, E., Mehdiyev, R., Blondal, K. and Altraja, A., 2018. Rapid tests reduce the burden of tuberculosis in Azerbaijan prisons: special emphasis on rifampicin-resistance. Revista espanola de sanidad penitenciaria, 20(3), p.111.

H Vargas González, H Gudiño Solorio, M Zghaib Rivero, D Molina Martínez, D Banderas Lares. HIV and tuberculosis in incarcerated patients in Mexico City during the 5 years, 2013 to 2018. J Int AIDS Soc. 2019 Apr; 22(Suppl Suppl 2): e25263.

Hanau-Bercot, B., Gremy, I., Raskine, L., Bizet, J., Gutierrez, M.C., Boyer-Mariotte, S.E.A., Bregeault, A., Lagrange, P.H. and Sanson Le Pors, M.J., 2000. A one-year prospective study (1994–1995) for a first evaluation of tuberculosis transmission in French prisons. The International Journal of Tuberculosis and Lung Disease, 4(9), pp.853-859.

Harries, A.D., Nyirenda, T.E., Yadidi, A.E., Gondwe, M.K., Kwanjana, J.H. and Salaniponi, F.M., 2004. Tuberculosis control in Malawian prisons: from research to policy and practice. The international journal of tuberculosis and lung disease, 8(5), pp.614-617

Hutton, M.D., Cauthen, G.M. and Bloch, A.B., 1993. Results of a 29-state survey of tuberculosis in nursing homes and correctional facilities. Public Health Reports, 108(3), p.305.

Ijaz, K., Yang, Z., Templeton, G., Stead, W.W., Bates, J.H. and Cave, M.D., 2004. Persistence of a strain of Mycobacterium tuberculosis in a prison system. The International Journal of Tuberculosis and Lung Disease, 8(8), pp.994-1000.

Kiter, G., Arpaz, S., Keskin, S., Sezgin, N., Budin, D. and Seref, O., 2003. Tuberculosis in Nazilli District Prison, Turkey, 1997–2001. The International Journal of Tuberculosis and Lung Disease, 7(2), pp.153-158.

Klopf, L.C., 1998. Tuberculosis control in the New York State Department of Correctional Services: a case management approach. American journal of infection control, 26(5), pp.534-537.

Koffi, N., Ngom, A.K., Aka-Danguy, E., Seka, A., Akoto, A. and Fadiga, D., 1997. Smear positive pulmonary tuberculosis in a prison setting: experience in the penal camp of Bouake, Ivory Coast. The International Journal of Tuberculosis and Lung Disease, 1(3), pp.250-253.

Mallick, G., Shewade, H.D., Agrawal, T.K., Kumar, A.M.V. and Chadha, S.S., 2017. Enhanced tuberculosis case finding through advocacy and sensitisation meetings in prisons of Central India. Public Health Action, 7(1), pp.67-70.

March, F., Coll, P., Guerrero, R.A., Busquets, E., Caylà, J.A. and Prats, G., 2000. Predictors of tuberculosis transmission in prisons: an analysis using conventional and molecular methods. Aids, 14(5), pp.525-535.

Martin, V., Guerra J, M., Cayla J, A., Rodriguez J, C., Blanco M, D. and Alcoba, M., 2001. Incidence of tuberculosis and the importance of treatment of latent tuberculosis infection in a Spanish prison population. The International Journal of Tuberculosis and Lung Disease, 5(10), pp.926-932.

McDaniel, C.J., Chitnis, A.S., Barry, P.M. and Shah, N., 2017. Tuberculosis trends in California correctional facilities, 1993–2013. The International Journal of Tuberculosis and Lung Disease, 21(8), pp.922-929.

Miller, T.L., Hilsenrath, P., Lykens, K., Mcnabb, S.J., Moonan, P.K. and Weis, S.E., 2006. Using cost and health impacts to prioritize the targeted testing of tuberculosis in the United States. Annals of epidemiology, 16(4), pp.305-312.

Nateniyom, S., Jittimanee, S.X., Ngamtrairai, N., Jittimanee, S., Boonpendetch, R., Moongkhetklang, V., Prapanwong, A., Rimwittayakorn, W., Pokaew, P., Aemdoung, K. and Pongpanit, S., 2004. Implementation of the DOTS strategy in prisons at provincial level, Thailand. The International Journal of Tuberculosis and Lung Disease, 8(7), pp.848-854.

Niero R. Risk of tuberculosis infection among imprisoned adults sao-paulo brazil prison 1976, 1977-1980. Bulletin of the International Union Against Tuberculosis 1982, 57(1): 36.

Noeske, J., Elo, G.A. and Mfondih, S.M., 2014. Tuberculosis incidence in Cameroonian prisons: a 1-year prospective study. South African Medical Journal, 104(3), pp.209-211.

Noeske, J., Ndi, N. and Mbondi, S., 2011. Controlling tuberculosis in prisons against confinement conditions: a lost case? Experience from Cameroon. The International journal of tuberculosis and lung disease, 15(2), pp.223-227.

Oliveira, L.G.D.D., Natal, S. and Camacho, L.A.B., 2015. Análise da implantação do Programa de Controle da Tuberculose em unidades prisionais no Brasil. Cadernos de Saúde Pública, 31, pp.543-554.

Paião, D.S.G., Lemos, E.F., Carbone, A.D.S.S., Sgarbi, R.V.E., Junior, A.L., da Silva, F.M., Brandão, L.M., Dos Santos, L.S., Martins, V.S., Simionatto, S. and Motta-Castro, A.R.C., 2016. Impact of mass-screening on tuberculosis incidence in a prospective cohort of Brazilian prisoners. BMC infectious diseases, 16(1), pp.1-8.

Rueda, Z.V., López, L., Vélez, L.A., Marín, D., Giraldo, M.R., Pulido, H., Orozco, L.C., Montes, F. and Arbeláez, M.P., 2013. High incidence of tuberculosis, low sensitivity of current diagnostic scheme and prolonged culture positivity in four Colombian prisons. A cohort study. PLoS One, 8(11), p.e80592

Rueda, Z.V., Arroyave, L., Marin, D., Lopez, L., Keynan, Y., Giraldo, M.R., Pulido, H. and Arbelaez, M.P., 2014. High prevalence and risk factors associated with latent tuberculous infection in two Colombian prisons. The International journal of tuberculosis and lung disease, 18(10), pp.1166-1171.

Sacchi, F.P., Praça, R.M., Tatara, M.B., Simonsen, V., Ferrazoli, L., Croda, M.G., Suffys, P.N., Ko, A.I., Andrews, J.R. and Croda, J., 2015. Prisons as reservoir for community transmission of tuberculosis, Brazil. Emerging infectious diseases, 21(3), p.452.

Slavuckij, A., Sizaire, V., Lobera, L., Matthys, F. and Kimerling, M.E., 2002. Decentralization of the DOTS programme within a Russian penitentiary system: How to ensure the continuity of tuberculosis treatment in pre-trial detention centres. The European Journal of Public Health, 12(2), pp.94-98.

Solera, J., Lopez, E., Serna, E., Vergara, L., Martinez-Alfaro, E. and Saez, L., 1993. Risk of tuberculosis in parenteral drug addicts with human immunodeficiency virus seropositivity. A cohort study in detoxication communities. Medicina clinica, 100(19), pp.725-729.

Stead, W.W., 1978. Undetected tuberculosis in prison: source of infection for community at large. Jama, 240(23), pp.2544-2547.

Toloba, Y., Ouattara, K., Soumaré, D., Kanouté, T., Berthé, G., Baya, B., Konaté, B., Keita, M., Diarra, B., Cissé, A. and Camara, F.S., 2018. Multidrug-resistant tuberculosis (MDR-TB) in a black African carceral area: Experience of Mali. Revue de pneumologie clinique, 74(1), pp.22-27.

Wong, M.Y., Leung, C.C., Tam, C.M., Kam, K.M., Ma, C.H. and Au, K.F., 2008. TB surveillance in correctional institutions in Hong Kong, 1999–2005. The international journal of tuberculosis and lung disease, 12(1), pp.93-98.

Yanjindulam, P., Oyuntsetseg, P., Sarantsetseg, B., Ganzaya, S., Amgalan, B., Narantuya, J., Nishikiori, N. and Lambregts-van Weezenbeek, C., 2012. Reduction of tuberculosis burden among prisoners in Mongolia: review of case notification, 2001–2010 [Notes from the field]. The International journal of tuberculosis and lung disease, 16(3), pp.327-329.

Zadeh, J.H., Nasehi, M., Rezaianzadeh, A., Tabatabaee, H., Rajaeifard, A. and Ghaderi, E., 2013. Pattern of reported tuberculosis cases in iran 2009–2010. Iranian journal of public health, 42(1), p.72.

References for the Outcome of Tuberculin and/or QuantiFERON Conversion

Arroyave, L., Keynan, Y., López, L., Marin, D., Arbeláez, M.P. and Rueda, Z.V., 2017. Negative latent tuberculosis at time of incarceration: identifying a very high-risk group for infection. Epidemiology & Infection, 145(12), pp.2491-2499.

Chigbu LN, Iroegbu CU, 2010. Incidence and spread of Mycobacterium tuberculosis-associated infection among Aba Federal prison inmates in Nigeria. Journal of health, population, and nutrition, 28(4), p.327.

de Oliveira RD, da Silva Santos A, Reis CB, de Cássia Leite A, Sacchi FP, de Araujo RC, Dos Santos PC, Rolla VC, Martinez L, Andrews J, Croda J. Primary Prophylaxis to Prevent Tuberculosis Infection in Prison Inmates: A Randomized, Double-Blind, Placebo-Controlled Trial. The American Journal of Tropical Medicine and Hygiene. 2020 Oct 7;103(4):1466-72.

Ferreira, M.M., Ferrazoli, L., Palaci, M., Salles, P.S., Medeiros, LA, Novoa, P, Kiefer, C.R., Schechtmann, M., Kritski, A.L., Johnson, WD, Riley, LW, 1996. Tuberculosis and HIV infection among female inmates in Sao Paulo, Brazil: a prospective cohort study. Journal of Acquired Immune Deficiency Syndromes, 13(2), pp.177-183.

Hung, R., 2003. Risk factors for tuberculosis conversion in a state prison. Portland, OR, USA: Scholar Archive, Oregon Health & Science University.

Levy, M.H., Butler, T.G. and Zhou, J., 2007. Prevalence of Mantoux positivity and annual risk of infection for tuberculosis in New South Wales prisoners, 1996 and 2001. New South Wales public health bulletin, 18(8), pp.119-124.

MacIntyre, C.R., Kendig, N., Kummer, L., Birago, S. and Graham, N.M., 1997. Impact of tuberculosis control measures and crowding on the incidence of tuberculous infection in Maryland prisons. Clinical Infectious Diseases, 24(6), pp.1060-1067.

Mamani, M., Mahmudian, H., Majzoobi, M.M. and Poorolajal, J., 2016. Prevalence and incidence rates of latent tuberculous infection in a large prison in Iran. The International Journal of Tuberculosis and Lung Disease, 20(8), pp.1072-1077.

Martin, V., Caylà, J.A., del Canto, M. and González, J., 2000. Incidence of tuberculous infection in a Spanish prison. Medicina clinica, 114(11), p.437.

Niero. Risk of tuberculosis infection among imprisoned adults sao paulo brazil prison 1976–1977 1980. *Bulletin of the International Union Against Tuberculosis* 57(1): 36 (1982).

Paião, D.S.G., Lemos, E.F., Carbone, A.D.S.S., Sgarbi, R.V.E., Junior, A.L., da Silva, F.M., Brandão, L.M., Dos Santos, L.S., Martins, V.S., Simionatto, S. and Motta-Castro, A.R.C., 2016. Impact of mass-screening on tuberculosis incidence in a prospective cohort of Brazilian prisoners. BMC infectious diseases, 16 (1), pp.1-8.

References for the Outcome of Prevalent Tuberculosis

Abrahão, R.M.C.M., Nogueira, P.A. and Malucelli, M.I.C., 2006. Tuberculosis in county jail prisoners in the western sector of the city of São Paulo, Brazil. The International Journal of Tuberculosis and Lung Disease, 10(2), pp.203-208.

Adane, K., Spigt, M., Ferede, S., Asmelash, T., Abebe, M. and Dinant, G.J., 2016. Half of pulmonary tuberculosis cases were left undiagnosed in prisons of the Tigray region of Ethiopia: implications for tuberculosis control. PloS one, 11(2), p.e0149453.

Addis, Z., Adem, E., Alemu, A., Birhan, W., Mathewos, B., Tachebele, B. and Takele, Y., 2015. Prevalence of smear positive pulmonary tuberculosis in Gondar prisoners, North West Ethiopia. Asian Pacific journal of tropical medicine, 8(2), pp.127-131.

Abebe, D.S., Bjune, G., Ameni, G., Biffa, D. and Abebe, F., 2011. Prevalence of pulmonary tuberculosis and associated risk factors in Eastern Ethiopian prisons. The International Journal of Tuberculosis and Lung Disease, 15(5), pp.668-673.

Aerts, A., Habouzit, M., Mschiladze, L., Malakmadze, N., Sadradze, N., Menteshashvili, O., Portaels, F. and Sudre, P., 2000. Pulmonary tuberculosis in prisons of the ex-USSR state Georgia: results of a nation-wide prevalence survey among sentenced inmates. The International Journal of Tuberculosis and Lung Disease, 4(12), pp.1104-1110.

Ali, S., Haileamlak, A., Wieser, A., Pritsch, M., Heinrich, N., Loscher, T., Hoelscher, M. and Rachow, A., 2015. Prevalence of pulmonary tuberculosis among prison inmates in Ethiopia, a cross-sectional study. PLoS One, 10(12), p.e0144040.

Alon, Y., 2015. The yield of tuberculosis screening of undocumented migrants from the Horn of Africa based on chest radiography.

Askarian, M., Karmi, A. and Sadeghi-Hassanaabadi, A., 2001. Tuberculosis among never-jailed drug abusers. Eastern Mediterranean Health Journal, 7, pp.461-464.

Assefzadeh, M., Barghi, R.G. and Shahidi, S.S., 2009. Tuberculosis case-finding and treatment in the central prison of Qazvin province, Islamic Republic of Iran. المجلة الصحية لشرق المتوسط، منظمة الصحة العالمية، المجلد الخامس.

Auregan, G., Rakotomanana, F., Ratsitorahina, M., Rakotoniaina, N., Rabemananjara, O., Raharimanana, R. and Boisier, P., 1995. Tuberculosis in the prison milieu at Antananarivo from 1990 to 1993. Archives de L'institut Pasteur de Madagascar, 62(1), pp.18-23.

Bah, H., Cisse, F.A., Camara, L.M., Diallo, O.H., Diallo, M. and Sow, O.Y., 2012. Prévalence de la tuberculose en milieu carcéral à Conakry, République de Guinée. La Revue de Médecine Légale, 3(4), pp.146-150.

Banda, H.T., Gausi, F., Harries, A.D. and Salaniponi, F.M., 2009. Prevalence of smear-positive pulmonary tuberculosis among prisoners in Malawi: a national survey. The international journal of tuberculosis and lung disease, 13(12), pp.1557-1559.

Banerjee, A., Harries, A.D., Mphasa, N., Yadid, A.E., Nyirenda, T. and Salaniponi, F.M., 2000. Prevalence of HIV, sexually transmitted disease and tuberculosis amongst new prisoners in a district prison, Malawi. Trop Doct, 30(1), pp.49-50.

Banu, S., Hossain, A., Uddin, M.K.M., Uddin, M.R., Ahmed, T., Khatun, R., Mahmud, A.M., Hyder, K.A., Lutfor, A.B., Karim, M.S. and Zaman, K., 2010. Pulmonary tuberculosis and drug resistance in Dhaka central jail, the largest prison in Bangladesh. PloS one, 5(5), p.e10759.

Bhatnagar, T., Ralte, M., Ralte, L., Sundaramoorthy, L. and Chhakchhuak, L., 2019. Intensified tuberculosis and HIV surveillance in a prison in Northeast India: Implementation research. Plos one, 14(7), p.e0219988.

Brock, N.N., Reeves, M., LaMarre, M. and Devoe, B., 1998. Tuberculosis case detection in a state prison system. Public Health Reports, 113(4), p.359.

Carbonara, S., Babudieri, S., Longo, B., Starnini, G., Monarca, R., Brunetti, B., Andreoni, M., Pastore, G., De Marco, V. and Rezza, G., 2005. Correlates of Mycobacterium tuberculosis infection in a prison population. European Respiratory Journal, 25(6), pp.1070-1076.

Carbone, A.D.S.S., Paião, D.S.G., Sgarbi, R.V.E., Lemos, E.F., Cazanti, R.F., Ota, M.M., Junior, A.L., Bampi, J.V.B., Elias, V.P.F., Simionatto, S. and Motta-Castro, A.R.C., 2015. Active and latent tuberculosis in Brazilian correctional facilities: a cross-sectional study. BMC infectious diseases, 15(1), pp.1-8.

Chevallay, B. and Bernheim, J., 1983. Epidemiology of pulmonary tuberculosis in the prison environment. Schweizerische medizinische Wochenschrift, 113(7), pp.261-265.

Chiang, C.Y., Hsu, C.J., Hsu, P.K., Suo, J. and Lin, T.P., 2002. Pulmonary tuberculosis in the Taiwanese prison population. Journal of the Formosan Medical Association, 101(8), pp.537-541.

Chigbu, L.N. and Iroegbu, C.U., 2010. Incidence and spread of Mycobacterium tuberculosis-associated infection among Aba Federal prison inmates in Nigeria. Journal of health, population, and nutrition, 28(4), p.327.

Crepet, A., Repetto, E., Al Rousan, A., Sané Schepisi, M., Girardi, E., Prestileo, T., Codecasa, L., Garelli, S., Corrao, S., Ippolito, G. and Decroo, T., 2016. Lessons learnt from TB screening in closed immigration centres in Italy. *International health*, *8*(5), pp.324-329.

Dememew, Z.G., Jerene, D., Datiko, D.G., Hiruy, N., Tadesse, A., Moile, T., Bekele, D., Yismawu, G., Melkieneh, K., Reshu, B. and Suarez, P.G., 2020. The yield of community-based tuberculosis and HIV among key populations in hotspot settings of Ethiopia: A cross-sectional implementation study. PloS one, 15(5), p.e0233730.

de Vries, G., Commandeur, S., Erkens, C., Haddad, W., Jansen, N., Kouw, P. and Kamphorst-Roemer, M., 2020. Towards selective tuberculosis screening of people in prison in a low-incidence country. European Respiratory Journal, 55(4).

Diendéré, E.A., Tiéno, H., Bognounou, R., Ouédraogo, D.D., Simporé, J., Ouédraogo-Traoré, R. and Drabo, J., 2011. Prevalence and risk factors associated with infection by human immunodeficiency virus, hepatitis B virus, syphilis and bacillary pulmonary tuberculosis in prisons in Burkina Faso. Medecine tropicale: revue du Corps de sante colonial, 71(5), p.464.

Dolla, C.K., Dhanraj, B., Malaisamy, M., Priyadarshini, C.P., Hissar, S.S., Natrajan, M., Krishnan, R. and Tripathy, S.P., 2019. Burden of pulmonary tuberculosis in modern prison: a cross sectional prevalence survey from south India. Indian Journal of Tuberculosis, 66(1), pp.189-192.

Estevan, A.O., Oliveira, S.M.D.V.L.D. and Croda, J., 2013. Active and latent tuberculosis in prisoners in the Central-West Region of Brazil. Revista da Sociedade Brasileira de Medicina Tropical, 46(4), pp.515-518.

Farhoudi, B., SeyedAlinaghi, S., Hosseini, M., Pahlaviani, F.G., Firouzeh, M.M., Shahbazi, M. and Mohraz, M., 2019. Prevalence of tuberculosis in a prison in Tehran by active case finding. Infectious Disorders-Drug Targets (Formerly Current Drug Targets-Infectious Disorders), 19(2), pp.167-170.

Fletcher. Chest x ray evaluation of ppd screening for tuberculosis in intravenous drug using detainees, American Review of Respiratory Disease 145(4 Part 2): A815 (1992).

Fuge, T.G. and Ayanto, S.Y., 2016. Prevalence of smear positive pulmonary tuberculosis and associated risk factors among prisoners in Hadiya Zone prison, Southern Ethiopia. BMC research notes, 9(1), p.201.

Gebrecherkos, T., Gelaw, B. and Tessema, B., 2016. Smear positive pulmonary tuberculosis and HIV co-infection in prison settings of North Gondar Zone, Northwest Ethiopia. BMC Public Health, 16(1), pp.1-10.

Gizachew Beza, M., Hunegnaw, E. and Tiruneh, M., 2017. Prevalence and associated factors of tuberculosis in prisons settings of East Gojjam Zone, Northwest Ethiopia. International journal of bacteriology, 2017.

Gray, N.J., Hansen-Knarhoi, M. and Krause, V.L., 2008. Tuberculosis in illegal foreign fishermen: whose public health are we protecting?. Medical Journal of Australia, 188(3), pp.144-147.

Guerra, J., Mogollón, D., González, D., Sanchez, R., Rueda, Z.V., Parra-López, C.A. and Murcia, M.I., 2019. Active and latent tuberculosis among inmates in La Esperanza prison in Guaduas, Colombia. PloS one, 14(1), p.e0209895.

Jamal, W., Azeemi, K., Waqar, M., Ikram, K., Zaidi, S.A. and Habib, S., 2019. Active case finding for tuberculosis among prisoners in Karachi, Pakistan.

Jittimanee, S.X., Ngamtrairai, N., White, M.C. and Jittimanee, S., 2007. A prevalence survey for smear-positive tuberculosis in Thai prisons. The International Journal of Tuberculosis and Lung Disease, 11(5), pp.556-561.

Jordan AM, Podewils LJ, Castro KG, Zishiri V, Charalambous S. Prevalence and risk factors of tuberculosis disease in South African correctional facilities in 2015. The International Journal of Tuberculosis and Lung Disease. 2019 Nov 1;23(11):1198-204.

Kalonji, G.M., De Connick, G., Ngongo, L.O., Nsaka, D.K., Kabengele, T., Kandolo, F.T., Ilunga-Ilunga, F., Adelin, A. and Giet, D., 2016. Prevalence of tuberculosis and associated risk factors in the Central Prison of Mbuji-Mayi, Democratic Republic of Congo. Tropical medicine and health, 44(1), p.30.

Karabela, S., Papaventsis, D., Georgoulas, S., Nikolaou, S., Ioannidis, P., Konstantinidou, E., Sainti, A., Marinou, I. and Kanavaki, S., 2010. Epidemiological monitoring of pulmonary tuberculosis in a correctional facility population, Athens, Greece, 2005-2009: P2080. Clinical Microbiology & Infection, 16.

Kazi, A.M., Shah, S.A., Jenkins, C.A., Shepherd, B.E. and Vermund, S.H., 2010. Risk factors and prevalence of tuberculosis, human immunodeficiency virus, syphilis, hepatitis B virus, and hepatitis C virus among prisoners in Pakistan. International Journal of Infectious Diseases, 14, pp.e60-e66.

King, K. and Vodicka, P., 2001. Screening for conditions of public health importance in people arriving in Australia by boat without authority. Medical journal of Australia, 175(11-12), pp.600-602.

Kuhleis, D., Ribeiro, A.W., Costa, E.R.D., Cafrune, P.I., Schmid, K.B., Costa, L.L.D., Ribeiro, M.O., Zaha, A. and Rossetti, M.L.R., 2012. Tuberculosis in a southern Brazilian prison. Memorias do Instituto Oswaldo Cruz, 107(7), pp.909-915.

Lasserre, B., 1986. Penitentiary medicine: an active type of medicine. Apropos of medical entrance examinations in the penitentiary environment. Revue medicale de la Suisse romande, 106(2), pp.173-179.

Layton, M.C., Henning, K.J., Alexander, T.A., Gooding, A.L., Reid, C., Heyman, B.M., Leung, J., Gilmore, D.M. and Frieden, T.R., 1997. Universal radiographic screening for tuberculosis among inmates upon admission to jail. American journal of public health, 87(8), pp.1335-1337.

Levy, M.H., Butler, T.G. and Zhou, J., 2007. Prevalence of Mantoux positivity and annual risk of infection for tuberculosis in New South Wales prisoners, 1996 and 2001. New South Wales public health bulletin, 18(8), pp.119-124.

Maggard KR, Hatwiinda S, Harris JB, Phiri W, Krüüner A, Kaunda K, Topp SM, Kapata N, Ayles H, Chileshe C, Henostroza G. Screening for tuberculosis and testing for human immunodeficiency virus in Zambian prisons. Bulletin of the World Health Organization. 2015;93:93-101.

Mamani, M., Mahmudian, H., Majzoobi, M.M. and Poorolajal, J., 2016. Prevalence and incidence rates of latent tuberculous infection in a large prison in Iran. The International Journal of Tuberculosis and Lung Disease, 20(8), pp.1072-1077.

Martín, S.V., Alvarez-Guisasola, Cayla, J.A. and Alvarez, J.L., 1995. Predictive factors of Mycobacterium tuberculosis infection and pulmonary tuberculosis in prisoners. International journal of epidemiology, 24(3), p.63.

Martin, V., Guerra J, M., Cayla J, A., Rodriguez J, C., Blanco M, D. and Alcoba, M., 2001. Incidence of tuberculosis and the importance of treatment of latent tuberculosis infection in a Spanish prison population. The International Journal of Tuberculosis and Lung Disease, 5(10), pp.926-932.

Martin, V., Gonzalez, P., Cayla, J.A., Mirabent, J., Cañellas, J., Pina, J.M. and Miret, P., 1994. Case-finding of pulmonary tuberculosis on admission to a penitentiary centre. Tubercle and Lung Disease, 75(1), pp.49-53. Centers for Disease Control and Prevention (CDC. Tuberculosis prevention in drug treatment centers and correctional facilities--selected US sites, 1990-1991. MMWR. Morbidity and mortality weekly report. 1993 Mar 26;42(11):210.

Merid, Y., Woldeamanuel, Y., Abebe, M., Datiko, D.G., Hailu, T., Habtamu, G., Assefa, G., Kempker, R.R., Blumberg, H.M. and Aseffa, A., 2018. High utility of active tuberculosis case finding in an Ethiopian prison. The International Journal of Tuberculosis and Lung Disease, 22(5), pp.524-529.

Moges, B., Amare, B., Asfaw, F., Tesfaye, W., Tiruneh, M., Belyhun, Y., Mulu, A. and Kassu, A., 2012. Prevalence of smear positive pulmonary tuberculosis among prisoners in North Gondar Zone Prison, northwest Ethiopia. BMC infectious diseases, 12(1), p.352.

Morasert, T., Worapas, W., Kaewmahit, R. and Uphala, W., 2018. Prevalence and risk factors associated with tuberculosis disease in Suratthani Central Prison, Thailand. The International Journal of Tuberculosis and Lung Disease, 22(10), pp.1203-1209.

Morishita, F., Garfin, A.M.C.G., Lew, W., Oh, K.H., Yadav, R.P., Reston, J.C., Infante, L.L., Acala, M.R.C., Palanca, D.L., Kim, H.J. and Nishikiori, N., 2017. Bringing state-of-the-art diagnostics to vulnerable populations: the use of a mobile screening unit in active case finding for tuberculosis in Palawan, the Philippines. PLoS One, 12(2), p.e0171310.

Navarro, P.D.D., Almeida, I.N.D., Kritski, A.L., Ceccato, M.D.G., Maciel, M.M.D., Carvalho, W.D.S. and Miranda, S.S.D., 2016. Prevalence of latent Mycobacterium tuberculosis infection in prisoners. Jornal Brasileiro de Pneumologia, 42(5), pp.348-355.

Nduaguba III, P., Brannan, G. and Shubrook, J., 2010. Evaluation of identifying tuberculosis infection and disease in a rural institutionalized population. Osteopathic Family Physician, 2(1), pp.10-13.

Niamatullah Kakar, F.A., Shafee, M. and Asmat, T., 2018. Study on Accuracy and Efficiency of Molecular Diagnostic Techniques used for Tuberculosis and Analysis of Associated Risk Factors for Tuberculosis in Jail Inmates of Quetta, Pakistan. Pakistan J. Zool, 50(4), pp.1461-1465.

Noeske, J., Ndi, N. and Mbondi, S., 2011. Controlling tuberculosis in prisons against confinement conditions: a lost case? Experience from Cameroon. The International journal of tuberculosis and lung disease, 15(2), pp.223-227.

Noeske, J., Kuaban, C., Amougou, G., Piubello, A. and Pouillot, R., 2006. Pulmonary tuberculosis in the central prison of Douala, Cameroon. East African medical journal, 83(1), pp.25-30.

Nogueira, P.A., Abrahão, R.M.C.D.M. and Galesi, V.M.N., 2012. Tuberculosis and latent tuberculosis in prison inmates. Revista de saude publica, 46(1), pp.119-127.

Nyangulu, D.S., Harries, A.D., Kang'Ombe, C., Yadidi, A.E., Chokani, K., Cullinan, T., Maher, D., Nunn, P. and Salaniponi, F.M., 1997. Tuberculosis in a prison population in Malawi. The Lancet, 350(9087), pp.1284-1287.

Nyirenda, T.E., Yadidi, A., Harries, A.D., Kwanjana, J. and Salanipon FL, 2000. Morbidity and mortality in prisons in Malawi. Tropical doctor, 30(2), pp.104-105

Öngen, G., Börekçi, Ş., İçmeli, Ö.S., Birgen, N., Karagül, G., Akgün, S., KILIÇASLAN, Z. and UMUT, S., 2013. Pulmonary tuberculosis incidence in Turkish prisons: importance of screening and case finding strategies. Tuberk Toraks, 61(1), pp.21-7.

Onu, E., Enejoh, V.A., Olarewaju, J., Igwegbe, L., Uwaezuoke, M., Igweike, P., Eze, C., Ugwu, E., Mpamugo, A., Mukiibi, M. and Olajide, I., 2017. What is the TB Burden in Nigerian Prisons?–An Enhanced TB Case Finding Program experience from 13 Nigerian Prisons. Annals of Global Health, 1(83), p.61.

Paião, D.S.G., Lemos, E.F., Carbone, A.D.S.S., Sgarbi, R.V.E., Junior, A.L., da Silva, F.M., Brandão, L.M., Dos Santos, L.S., Martins, V.S., Simionatto, S. and Motta-Castro, A.R.C., 2016. Impact of mass-screening on tuberculosis incidence in a prospective cohort of Brazilian prisoners. BMC infectious diseases, 16(1), pp.1-8.

Pedro, H.D.S.P., Nardi, S.M.T., Pereira, M.I.F., Goloni, M.D.R.A., Pires, F.C., Tolentino, F.M., Oliveira, R.S. and Rossit, A.R.B., 2011. Mycobacterium tuberculosis detection in the penitentiary system. Revista de Patologia Tropical/Journal of Tropical Pathology, 40(4), pp.287-296.

Pelissari, D.M., Kuhleis, D.C., Bartholomay, P., Barreira, D., Oliveira, C.L.P., de Jesus, R.S., Possa, L.A., Jarczewski, C.A., Nemeth, L.T., de Araujo, N.D. and Alves, P.B.L., 2018. Prevalence and screening of active tuberculosis in a prison in the South of Brazil. The International Journal of Tuberculosis and Lung Disease, 22(10), pp.1166-1171.

Pendzich, J., Maksymowicz-Mazur, W., Pawłowska, J., Filipczyk, Ł., Kulawik, I., Zientek, J. and Kozielski, J., 2015. Tuberculosis among the homeless and inmates kept in custody and in penitentiary institutions in the Silesia region. Advances in Respiratory Medicine, 83(1), pp.23-29.

Prasad, B.M., Thapa, B., Chadha, S.S., Das, A., Babu, E.R., Mohanty, S., Pandurangan, S. and Tonsing, J., 2017. Status of tuberculosis services in Indian prisons. International Journal of Infectious Diseases, 56, pp.117-121.

Puisis, M., Feinglass, J., Lidow, E. and Mansour, M., 1996. Radiographic screening for tuberculosis in a large urban county jail. Public Health Reports, 111(4), p.330.

Rao, N.A., 2004. Prevalence of pulmonary tuberculosis in Karachi central prison. Journal – Pakistan Medical Association, 54(8), pp.413-414.

Risser, W.L. and Smith, K.C., 2005. Tuberculosis in incarcerated youth in Texas. JAMA, 293(22), pp.2713-2717.

Ritter, C. and Elger, B.S., 2012. Prevalence of positive tuberculosis skin tests during 5 years of screening in a Swiss remand prison. The International journal of tuberculosis and lung disease, 16(1), pp.65-69.

Salazar-De La Cuba, A.L., Ardiles-Paredes, D.F., Araujo-Castillo, R.V. and Maguiña, J.L., 2019. High prevalence of self-reported tuberculosis and associated factors in a nation-wide census among prison inmates in Peru. Tropical Medicine & International Health, 24(3), pp.328-338.

Sanchez, A., Larouzé, B., Espinola, A.B., Pires, J., Capone, D., Gerhardt, G., Cesconi, V., Procopio, M.J., Hijjar, M. and Massari, V., 2009. Screening for tuberculosis on admission to highly endemic prisons? The case of Rio de Janeiro State prisons. The International journal of tuberculosis and lung disease, 13(10), pp.1247-1252.

Sanchez, A., Massari, V., Gerhardt, G., Espinola, A.B., Siriwardana, M., Camacho, L.A.B. and Larouzé, B., 2013. X ray screening at entry and systematic screening for the control of tuberculosis in a highly endemic prison. BMC public health, 13(1), p.983.

Sanchez, A., Gerhardt, G., Natal, S., Capone, D., Espinola, A., Costa, W., Pires, J., Barreto, A., Biondi, E. and Larouzé, B., 2005. Prevalence of pulmonary tuberculosis and comparative evaluation of screening strategies in a Brazilian prison. The International Journal of Tuberculosis and Lung Disease, 9(6), pp.633-639.

Sánchez, A.M., Villena, L.M., Castro, J.R., Aguayo, M.C. and de la Llave Pujol, E., 1997, December. Study of tuberculosis in Huelva prison. In Anales de medicina interna (Madrid, Spain: 1984) (Vol. 14, No. 12, pp. 607-610).

Santos, Andrea da Silva, Roberto Dias de Oliveira, Everton Ferreira Lemos, Fabiano Lima, Ted Cohen, Olivia Cords, Leonardo Martinez et al. "Yield, Efficiency and Costs of Mass Screening Algorithms for Tuberculosis in Brazilian Prisons." Clinical Infectious Diseases (2020).

Saunders DL, Olive DM, Wallace SB, Lacy D, Leyba R, Kendig NE. Tuberculosis screening in the federal prison system: an opportunity to treat and prevent tuberculosis in foreign-born populations. Public Health Reports. 2016 Nov 30.

Schneider, D.L. and Lobato, M.N., 2007. Tuberculosis control among people in US Immigration and Customs Enforcement custody. American journal of preventive medicine, 33(1), pp.9-14.

Séri, B., Koffi, A., Danel, C., Ouassa, T., Blehoué, M.A., Ouattara, E., Assemien, J.D.A., Masumbuko, J.M., Coffie, P., Cartier, N. and Laurent, A., 2017. Prevalence of pulmonary tuberculosis among prison inmates: A cross-sectional survey at the Correctional and Detention Facility of Abidjan, Côte d'Ivoire. PloS one, 12(7), p.e0181995.

Shah, S.A., Mujeeb, S.A., Mirza, A., Nabi, K.G. and Siddiqui, Q., 2003. Prevalence of pulmonary tuberculosis in Karachi juvenile jail, Pakistan. EMHJ-Eastern Mediterranean Health Journal, 9 (4), 667-674, 2003.

Shah, S., Ali, M.O.M.I.N.A., Ahmad, M.U.S.H.T.A.Q. and Hamadan, U., 2013. Screening of jail inmates for HIV and tuberculosis. Pak J Med Health Sci, 7(1), p.13Á19

Singh, S., Prasad, R. and Mohanty, A., 1999. High prevalence of sexually transmitted and blood-borne infections amongst the inmates of a district jail in Northern India. International journal of STD & AIDS, 10(7), pp.475-478.

Slavuckij, A., Sizaire, V., Lobera, L., Matthys, F. and Kimerling, M.E., 2002. Decentralization of the DOTS programme within a Russian penitentiary system: How to ensure the continuity of tuberculosis treatment in pre-trial detention centres. The European Journal of Public Health, 12(2), pp.94-98.

Sretrirutchai, S., Silapapojakul, K., Palittapongarnpim, P., Phongdara, A. and Vuddhakul, V., 2002. Tuberculosis in Thai prisons: magnitude, transmission and drug susceptibility. The International Journal of Tuberculosis and Lung Disease, 6(3), pp.208-214.

Swartz, J.A., 2011. Chronic medical conditions among jail detainees in residential psychiatric treatment: a latent class analysis. Journal of Urban Health, 88(4), pp.700-717.

Takashima, H.T., Cruess, D.F., McDonald, K.R., Duggirala, S. and Gaydos, J.C., 1996. Tuberculosis and HIV infection in new inmates in Federal Bureau of Prisons facilities. Military medicine, 161(5), pp.265-267.

Telisinghe, L., Fielding, K.L., Malden, J.L., Hanifa, Y., Churchyard, G.J., Grant, A.D. and Charalambous, S., 2014. High tuberculosis prevalence in a South African prison: the need for routine tuberculosis screening. PloS one, 9(1), p.e87262.

Toloba, Y., Soumaré, D., Ouattara, K., Kanouté, T., Boré, O., Dolo, O., Baya, B., Berthé, G. and Diallo, S., 2017. Respiratory diseases in black African carceral area. Revue des maladies respiratoires, 34(7), pp.729-733.

Tong, Y., Jiang, S., Guan, X., Hou, S., Cai, K., Tong, Y., Cai, L., Liu, J. and Lu, Q., 2019. Epidemic Situation of Tuberculosis in Prisons in the Central Region of China. The American journal of tropical medicine and hygiene, 101(3), pp.510-512.

Tsegaye Sahle, E., Blumenthal, J., Jain, S., Sun, S., Young, J., Manyazewal, T., Woldeamanuel, H., Teferra, L., Feleke, B., Vandenberg, O. and Rey, Z., 2019. Bacteriologically-confirmed pulmonary tuberculosis in an Ethiopian prison: Prevalence from screening of entrant and resident prisoners. Plos one, 14(12), p.e0226160.

Wali, A., Khan, D., Safdar, N., Shawani, Z., Fatima, R., Yaqoob, A., Qadir, A., Ahmed, S., Rashid, H., Ahmed, B. and Khan, S., 2019. Prevalence of tuberculosis, HIV/AIDS, and hepatitis; in a prison of Balochistan: a cross-sectional survey. BMC public health, 19(1), pp.1-8.

Vieira, A.A., Ribeiro, S.A., Siqueira, A.M.D., Galesi, V., dos Santos, L.A. and Golub, J.E., 2010. Prevalence of patients with respiratory symptoms through active case finding and diagnosis of pulmonary tuberculosis among prisoners and related predictors in a jail in the city of Carapicuíba, Brazil. Revista Brasileira de Epidemiologia, 13, pp.641-650.

Wang, E.A., McCrann, C.H., Notha, M. and Mwasekaga, M.J., 2003. Rapid assessment of tuberculosis in a large prison system-Botswana, 2002. MMWR: Morbidity & Mortality Weekly Report, 52(12), pp.250-250.

White, M.C., Tulsky, J.P., Portillo, C.J., Menendez, E., Cruz, E. and Goldenson, J., 2001. Tuberculosis prevalence in an urban jail: 1994 and 1998. The International Journal of Tuberculosis and Lung Disease: the Official Journal of the International Union Against Tuberculosis and Lung Disease, 5(5), pp.400-404.

Winetsky, D.E., Almukhamedov, O., Pulatov, D., Vezhnina, N., Dooronbekova, A. and Zhussupov, B., 2014. Prevalence, risk factors and social context of active pulmonary tuberculosis among prison inmates in Tajikistan. PLoS One, 9(1), p.e86046.

Wong, M.Y., Leung, C.C., Tam, C.M., Kam, K.M., Ma, C.H. and Au, K.F., 2008. TB surveillance in correctional institutions in Hong Kong, 1999–2005. *The international journal of tuberculosis and lung disease*, *12*(1), pp.93-98.

Yates S, Story A, Hayward A. Screening prisoners for tuberculosis: what should the UK do? Thorax, 64 (2009), p. A105.

Zhang (2012). Analysis of the findings of tuberculosis patients in a detention center in Lanzhou City.

Zishiri, V., Charalambous, S., Shah, M.R., Chihota, V., Page-Shipp, L., Churchyard, G.J. and Hoffmann, C.J., 2015, January. Implementing a large-scale systematic tuberculosis screening program in correctional facilities in South Africa. In Open forum infectious diseases (Vol. 2, No. 1). Oxford University Press.

5. Supplementary Table 1. Multivariable Bayesian meta-regression analysis of prevalent and incident tuberculosis among prisoners from studies included in the systematic review.

**Characteristic**	**Odds Ratio (95% CI)**	**Incidence Rate Ratio (95% CI)**
Study Design		
Registry-based notification	1 (Referent)	1 (Referent)
Study-based	0.7 (0.1–2.7)	0.5 (0.2–1.0)
Data Collection, Years†		
1970s/1980s	1 (Referent)	1 (Referent)
1990s	1.2 (0.3–3.3)	2.6 (0.7–6.8)
2000s	0.8 (0.2–2.1)	1.8 (0.5–4.8)
2010s	0.7 (0.2–1.8)	1.7 (0.4–4.6)
Global Region*		
North America	1 (Referent)	1 (Referent)
South America	8.5 (2.2–22.7)	29.0 (10.4–66.0)
Europe	3.7 (1.1–9.3)	15.1 (6.1–31.6)
Africa	7.7 (2.3–19.1)	50.3 (13.8–133.7)
South-East Asia	8.3 (1.9–24.3)	48.3 (6.9–180.8)
Western Pacific	3.5 (2.8–10.1)	15.4 (3.2–46.4)
Eastern Mediterranean	5.0 (1.2–14.1)	11.2 (1.8–37.9)

Abbreviations: CI, credible intervals.

† Due to low number of tuberculosis incidence and prevalence studies in the 1970's and 1980's, we grouped these two decades into one group.

* The World Health Organization classifies the Americas as one region – due to substantial differences in tuberculosis burden among incarcerated populations in North and South America, we separated out this region.

Description of Results from Table.

In multivariable meta-regression, the global region of the study was associated with a study's prevalence of tuberculosis (Table 3). Compared to studies in North America, studies in South America (Adjusted OR [AOR], 11.3; 95% CI, 2.8–32.1), Africa (AOR, 9.9; 95% CI, 2.9–25.8), South-East Asia (AOR, 8.9; 95% CI, 1.4–31.4), the Eastern Mediterranean (AOR, 12.6; 95% CI, 2.6–39.6), and Europe (AOR, 6.7; 95% CI, 1.8–18.3) were at substantially higher risk of prevalent tuberculosis. Other variables included in the meta-regression were not statistically related to prevalent tuberculosis, including year of study implementation and the study design.

6. Supplementary Table 2. Distribution of articles by languages.

Languages	n
French	7
Portuguese	3
Russian	1
Spanish	6
Chinese	1

7. Supplementary Table 3. Checklist of items to include when reporting a systematic review or meta-analysis

Section/topic	#	Checklist item	Reported on page #
**TITLE**			
Title	1	Identify the report as a systematic review, meta-analysis, or both.	1
**ABSTRACT**			
Structured summary	2	Provide a structured summary including, as applicable: background; objectives; data sources; study eligibility criteria, participants, and interventions; study appraisal and synthesis methods; results; limitations; conclusions and implications of key findings; systematic review registration number.	3-4
**INTRODUCTION**			
Rationale	3	Describe the rationale for the review in the context of what is already known.	5
Objectives	4	Provide an explicit statement of questions being addressed with reference to participants, interventions, comparisons, outcomes, and study design (PICOS).	5
**METHODS**			
Protocol and registration	5	Indicate if a review protocol exists, if and where it can be accessed (e.g., Web address), and, if available, provide registration information including registration number.	6
Eligibility criteria	6	Specify study characteristics (e.g., PICOS, length of follow-up) and report characteristics (e.g., years considered, language, publication status) used as criteria for eligibility, giving rationale.	7
Information sources	7	Describe all information sources (e.g., databases with dates of coverage, contact with study authors to identify additional studies) in the search and date last searched.	6
Search	8	Present full electronic search strategy for at least one database, including any limits used, such that it could be repeated.	6
Study selection	9	State the process for selecting studies (i.e., screening, eligibility, included in systematic review, and, if applicable, included in the meta-analysis).	6-7
Data collection process	10	Describe method of data extraction from reports (e.g., piloted forms, independently, in duplicate) and any processes for obtaining and confirming data from investigators.	6-7
Data items	11	List and define all variables for which data were sought (e.g., PICOS, funding sources) and any assumptions and simplifications made.	6-8

Section/topic	#	Checklist item	Reported on page #
Risk of bias in individual studies	12	Describe methods used for assessing risk of bias of individual studies (including specification of whether this was done at the study or outcome level), and how this information is to be used in any data synthesis.	8
Summary measures	13	State the principal summary measures (e.g., risk ratio, difference in means).	8-9
Synthesis of results	14	Describe the methods of handling data and combining results of studies, if done, including measures of consistency (e.g., I^2) for each meta-analysis.	8-10
Risk of bias across studies	15	Specify any assessment of risk of bias that may affect the cumulative evidence (e.g., publication bias, selective reporting within studies).	8
Additional analyses	16	Describe methods of additional analyses (e.g., sensitivity or subgroup analyses, meta-regression), if done, indicating which were pre-specified.	9-10
**RESULTS**			
Study selection	17	Give numbers of studies screened, assessed for eligibility, and included in the review, with reasons for exclusions at each stage, ideally with a flow diagram.	11
Study characteristics	18	For each study, present characteristics for which data were extracted (e.g., study size, PICOS, follow-up period) and provide the citations.	11
Risk of bias within studies	19	Present data on risk of bias of each study and, if available, any outcome-level assessment (see Item 12).	11
Results of individual studies	20	For all outcomes considered (benefits or harms), present, for each study: (a) simple summary data for each intervention group and (b) effect estimates and confidence intervals, ideally with a forest plot.	11-13
Synthesis of results	21	Present results of each meta-analysis done, including confidence intervals and measures of consistency.	11-13
Risk of bias across studies	22	Present results of any assessment of risk of bias across studies (see Item 15).	11
Additional analysis	23	Give results of additional analyses, if done (e.g., sensitivity or subgroup analyses, meta-regression [see Item 16]).	13
**DISCUSSION**			
Summary of evidence	24	Summarize the main findings including the strength of evidence for each main outcome; consider their relevance to key groups (e.g., health care providers, users, and policy makers).	14-16

Section/topic	#	Checklist item	Reported on page #
Limitations	25	Discuss limitations at study and outcome level (e.g., risk of bias), and at review level (e.g., incomplete retrieval of identified research, reporting bias).	15-16
Conclusions	26	Provide a general interpretation of the results in the context of other evidence, and implications for future research.	16
**FUNDING**			
Funding	27	Describe sources of funding for the systematic review and other support (e.g., supply of data); role of funders for the systematic review.	20

8. Supplementary Table 4. Study characteristics for studies included in the incidence of tuberculosis infection outcome

First Author	Publication Year	Country	No. Prisons	Facility Type	Global Region	Prison Control measures
Niero	1982	Brazil	1	Prison	South America	Not reported
Ferreira	1996	Brazil	1	Prison	South America	Not reported
MacIntyre	1997	United States	16	Prison	North America	Annual mass screenings, Entrance screenings, Isolation for diagnosed TB cases, administration of isoniazid prophylaxis
Martin	2000	Spain	1	Prison	European	Not reported
Hung	2003	United States	13	Prison, Jail	North America	Annual mass screenings, isolation, preventative therapy, contact investigation
Levy	2007	Australia	29	Prison	Western Pacific	Not reported
Chigbu	2010	Nigeria	1	Prison	African	Not reported
Paião	2016	Brazil	12	Prison	South America	Not reported
Mamani	2016	Iran	1	Prison	Eastern Mediterranean	Not reported
Arroyave	2017	Colombia	2	Prison	South America	Not reported
Oliviera	2020	Brazil	3	Prison	South America	Annual mass screenings

9. Supplementary Table 5. Study characteristics for studies included in the incidence of tuberculosis outcome

First Author	Publication Year	Country	No. Prisons	Facility Type	Global Region	Prison Control measures
Yanjindulam	2012	Mongolia	39	Combination of detention centers	Western Pacific	Entrance screenings, DOTS implementation
de Oliveira	2015	Brazil		Prison	South America	Annual mass screenings, Entrance & Active screenings for 2 prisons only
de Oliveira	2004	Brazil		Prison	South America	Not reported
Zambrano	2017	Honduras		Prison	South America	Not reported
Wong	2008	China	24	Combination of detention centers	Western Pacific	Entrance screenings, Isolation for diagnosed TB cases
Toloba	2018	Mali	1	Prison	African	None
Stead	1989	United States	2	Prison	North America	Not reported
Solera	1993	Spain	2	Drug detention center	European	Not reported
Slavuckij	2002	Russia	1	Jail	European	Entrance screenings, Screened once every 6 months
Schwitters	2014	Uganda	10	Prison	African	Annual mass screenings, Entrance screenings
Sacchi	2015	Brazil	1	Prison	South America	Not reported
Rueda	2013	Colombia	2	Prison	South America	Not reported
Paião	2016	Brazil	12	Prison	South America	Not reported
Noeske	2014	Cameroon	1	Prison	African	Entrance screenings, Annual mass screenings in 4 of prisons
Noeske	2011	Cameroon	N/A	Prison	African	Entrance screenings, Peer education, contact tracing
Niero	1982	Brazil	1	Prison	South America	Not reported
Nateniyom	2004	Thailand	16	Prison	South-East Asian	Ambulatory unit, passive detection, treatment
Mor	2008	Israel	19	Prison	European	Isolation for diagnosed TB cases
Miri	2014	Iran	N/A	Prison	Eastern Mediterranean	Not reported
Miller	2006	United States	1	Jail	North America	Annual mass & entrance screenings
McDaniel	2017	United States	N/A	Prison	North America	Not reported
Martin	2001	Spain	1	Prison	European	Not reported
March	2000	Spain	5	Prison	European	Entrance screenings

Mallick	2017	India	N/A	Prison	South-East Asian	None
MacNeil	2005	United States	N/A	Combination of detention centers	North America	Not reported
Lambert	2016	United States	N/A	Combination of detention centers	North America	Not reported
Koffi	1997	Cote d'Ivoire	1	Prison	African	Not reported
Klopf	1998	United States	N/A	Prison	North America	Annual mass screenings, Isolation for diagnosed TB cases, Contact investigations, TST, TB educational material
Kiter	2003	Turkey	1	Prison	European	Entrance & annual mass screenings, Isolation for diagnosed TB cases
Kawatsu	2015	Japan	N/A	Prison	Western Pacific	Annual mass screenings
Ijaz	2004	United States	19	Prison	North America	Entrance & annual mass screenings, Isolation for diagnosed TB cases, LTBI testing at new admission. Chest radiographs for those with symptoms, HIV positivity, a history of TB, or documented TST+
Hutton	1993	United States	N/A	Combination of detention centers	North America	Not reported
Hasan Zadeh	2013	Iran	N/A	Prison	Eastern Mediterranean	Not reported
Harries	2004	Malawi	42	Prison	African	Not reported
Hanau-Berçot	2000	France	10	Prison	European	Entrance screenings, Isolation for diagnosed TB cases, self-reported symptomatology
Fountain	1997	United States	3	Combination of detention centers	North America	Not reported
Fernandez de la Hoz	2001	Spain	9	Prison	European	Not reported
Ferreira	1996	Brazil	1	Prison	South America	Not reported
Dhuria	2016	India	1	Combination of detention centers	South-East Asian	Entrance screenings, Quarantine in TB ward for male inmates
Degner	2016	United States	7	Jail	North America	Entrance screenings, isolation of active/suspected patients

Costa-Junior	2016	Brazil	2	Prison	South America	Not reported
Cioran	2013	Romania	N/A	Prison	European	Not reported
Chaves	1993	Spain	N/A	Prison	European	Not reported
Chaves	1997	Spain	7	Prison	European	Not reported
Chigbu	2010	Nigeria	1	Prison	African	Not reported
Castañeda-Hernández	2013	Colombia	1	Prison	South America	Not reported
Bubochkin	1995	Russia	N/A	Prison	European	Not reported
Braun	1989	United States	52	Prison	North America	Entrance screenings, TSTs at prison entry
Bourdillon	2017	Brazil	N/A	Prison	South America	Not reported
Bock	1998	United States	138	Combination of detention centers	North America	Entrance screenings, Isolation for diagnosed TB cases
Biljana	2013	Macedonia	N/A	Prison	European	Not reported
Baillargeon	2002	United States	N/A	Prison	North America	Annual mass screenings, Treatment and monthly sputum smears/cultures; respiratory isolation
Ayala	2016	El Salvador	25	Prison	South America	education of prison health staff prevention and treatment - DOTS, biannual screening, inmate volunteer network
Auregan	1995	Madagascar	1	Prison	African	Not reported
Alavi	2014	Iran	N/A	Prison	Eastern Mediterranean	Not reported
Aguilera	2016	Chile	46	Prison	South America	Entrance & periodic mass screenings
Aerts	2006	10 countries	N/A	Combination of detention centers	European	Entrance screenings, Isolation for diagnosed TB patients

10. Supplementary Table 6. Study characteristics for studies included in the prevalence of tuberculosis outcome

First Author	Publication Year	Country	No. Prisons	Facility Type	Global Region	Prison Control measures
Abebe	2011	Ethiopia	3	Prison	African	Not reported
Abrahão	2006	Brazil	9	Jail	South America	Not reported
Adane	2016	Ethiopia	9	Prison	African	Not reported
Addis	2015	Ethiopia	1	Prison	African	Not reported
Aerts	2000	Georgia	12	Prison	European	Not reported
Ali	2015	Ethiopia	13	Prison	African	Not reported
Askarian	2001	Iran	1	Prison	Eastern Mediterranean	Not reported
Assefzadeh	2009	Iran	1	Prison	Eastern Mediterranean	Ventilation
Auregan	1995	Madagascar	1	Prison	African	Not reported
Bah	2012	Guinea	1	Prison	African	Not reported
Banda	2009	Malawi	18	Prison	African	Entrance & Periodic screenings n
Banerjee	2000	Malawi	1	Prison	African	Not reported
Banu	2010	Bangladesh	1	Jail	South-East Asian	None
Beza	2017	Ethiopia	3	Prison	African	Not reported
Bock	1998	United States	138	Combination of detention centers	North America	Entrance screenings, Isolation for diagnosed TB cases
Carbonara	2005	Italy	9	Prison	European	Not reported
Chevallay	1983	Switzerland	1	Prison	European	Not reported
Chiang	2002	Taiwan	29	Combination of detention centers	Western Pacific	Not reported
Crepet	2016	Italy	4	Immigration detention center	European	Not reported
Chigbu		Nigeria				Not reported
Diendere	2011	Burkina Faso	1	Prison	African	Not reported
Estevan	2013	Brazil	1	Jail	South America	Not reported
Fletcher	1992	United States	1	Prison	North America	Not reported
Fuge	2016	Ethiopia	1	Prison	African	Not reported
Gebrecherkos	2016	Ethiopia	1	Prison	African	Not reported
Gray	2008	Australia	1	Immigration detention center	Western Pacific	Entrance screenings

Jittimanee	2007	Thailand	27	Prison	South-East Asian	Not reported
Kalonji	2016	Democratic Republic of Congo	1	Prison	African	Not reported
Karabela	2010	Greece	1	Prison	European	Not reported
Kazi	2010	Pakistan	1	Combination of detention centers	Eastern Mediterranean	Not reported
King	2001	Australia	3	Immigration detention center	Western Pacific	Entrance screenings
Kuhleis	2012	Brazil	1	Prison	South America	Not reported
Lasserre	1986	Switzerland	2	Prison	European	Entrance screenings
Layton	1997	United States	1	Jail	North America	Entrance screenings
Levy	2007	Australia	29	Prison	Western Pacific	Not reported
Maggard	2015	Zambia	6	Prison	African	TB Isolation
Mamani	2016	Iran	1	Prison	Eastern Mediterranean	Not reported
Martin	2001	Spain	1	Prison	European	Not reported
Martin	1994	Spain	1	Prison	European	Not reported
Merid	2018	Ethiopia	1	Prison	African	Not reported
Moges	2012	Ethiopia	1	Prison	African	Not reported
Mor	2015	Israel	1	Immigration detention center	European	Entrance screenings
Morishita	2017	Philippines	6	Combination of detention centers	Western Pacific	Not reported
Nduaguba	2010	United States	1	Jail	North America	Not reported
Noeske	2011	Cameroon	1	Prison	African	Entrance screenings, Peer education, contact tracing
Noeske	2006	Cameroon	1	Prison	African	None
Nogueira	2012	Brazil	2	Combination of detention centers	South America	Not reported
Nyangulu	1997	Malawi	1	Prison	African	Not reported
Nyirenda	2000	Malawi	7	Prison	African	Not reported
Ongen	2013	Turkey	10	Prison	European	Not reported
Onu	2017	Nigeria	13	Prison	African	Not reported
Paião	2016	Brazil	12	Prison	South America	Not reported
Paro Pedro	2011	Brazil	3	Prison	South America	Not reported
Pendzich	2015	Poland	7	Combination of detention centers	European	Not reported
Prasad	2017	India	61	Prison	South-East Asian	Not reported
Puisis	1996	United States	1	Jail	North America	Entrance screenings

Rao	2004	Pakistan	1	Prison	Eastern Mediterranean	Not reported
Risser	2005	United States	5	Juvenile detention center	North America	Entrance screenings
Ritter	2012	Switzerland	1	Jail	European	Entrance screenings
Sanchez	1995	Spain	1	Prison	European	Not reported
Sanchez	2013	Brazil	1	Prison	South America	None
Sanchez	2009	Brazil	3	Prison	South America	Not reported
Sanchez	2005	Brazil	1	Prison	South America	None
Sanchez Moreno	1997	Spain	1	Prison	European	None
Santos Carbone	2015	Brazil	12	Prison	South America	Not reported
Saunders	2001	United States	1	Jail	North America	Entrance & Annual mass screenings, Isolation for diagnosed TB cases
Schneider	2007	United States	15	Immigration detention center	North America	Entrance screenings
Seri	2017	Cote d'Ivoire	1	Prison	African	Entrance screenings
Shah	2013	Pakistan	1	Jail	Eastern Mediterranean	Not reported
Shah	2003	Pakistan	1	Prison	Eastern Mediterranean	Not reported
Singh	1999	India	1	Combination of detention centers	South-East Asian	Not reported
Slavuckij	2002	Russia	1	Jail	European	Entrance screenings, Screened once every 6 months
Sretrirutchai	2002	Thailand	1	Prison	South-East Asian	Not reported
Swartz	2011	United States	1	Psychiatric Facility	North America	Not reported
Takashima	1996	United States	69	Prison	North America	Entrance screenings
Telisinghe	2014	South Africa	1	Prison	African	Entrance screenings
Toloba	2017	Mali	1	Prison	African	No contact/mass screening
Vieira	2010	Brazil	1	Jail	South America	Not reported
Wang	2003	Botswana	4	Prison	African	None
White	2001	United States	1	Jail	North America	Entrance screenings
Winetsky	2014	Tajikistan	2	Prison	European	Annual mass screenings
Wong	2008	China	24	Combination of detention centers	Western Pacific	Entrance screenings, Isolation for diagnosed TB cases

Yates	2009	United Kingdom	N/A	Prison	European	Entrance screenings
Ye Qing	2019	China	2	Prison	Western Pacific	Not reported
Zhang	2012	China	1	Detention Center	Western Pacific	Isolation for diagnosed TB cases, UV disinfection, TB patients wearing masks, disinfecting with peroxyacetic acid
Zishiri	2014	South Africa	4	Prison	African	Entrance & Annual mass screenings
de Navarro	2016	Brazil	2	Prison	South America	Not reported

11. Supplementary Table 5. Study quality of included studies.

	**Incident infection**	**Incident tuberculosis**	**Prevalent tuberculosis**
Quality of Study			
High	8 (73)	7 (13.7)	36 (34.0)
Moderate	3 (27)	20 (39.2)	57 (53.8)
Low	0 (0)	24 (47.1)	13 (12.3)

12. Study Quality Assessment

Representativeness

Representativeness of the sample (size)

A* - initially screened >= 70% of inmates;
B - initially screened <70% of inmates
C Percentage of prisoners screened is not specified

Representativeness of the sample (subset)

A*- Cohort representative of prison(s)' population (no subset)
B- Selected subset of prisoners (HIV/IDU etc..)
C- The derivation of the cohort is not specified

Selection

Exposure status

A * - measured person-years of exposure.
B - no reporting of person-time exposure. (Case notifications.)

Demonstration that incident tuberculosis infection/disease not present at baseline

A*- Reported testing for and numbers of tuberculosis cases at baseline and excluded coprevalent cases.
B*- Reported coprevalent tuberculosis as an exclusion criteria for incident tuberculosis.
C - Number of coprevalent cases not excluded or not reported

Comparability

Case definition

A* - Case definition stated explicitly
B - No case definition given

Outcome

Assessment of tuberculosis - tests available (2 total points) - can be A/B* because not mutually exclusive*

A*- Microbiological testing
B* Radiographical and clinical (must have both);
C Radiographical or clinical;
D No description

13. Supplementary Figure 1. Availability of study-level data from meta-analysis on tuberculosis among prisons.

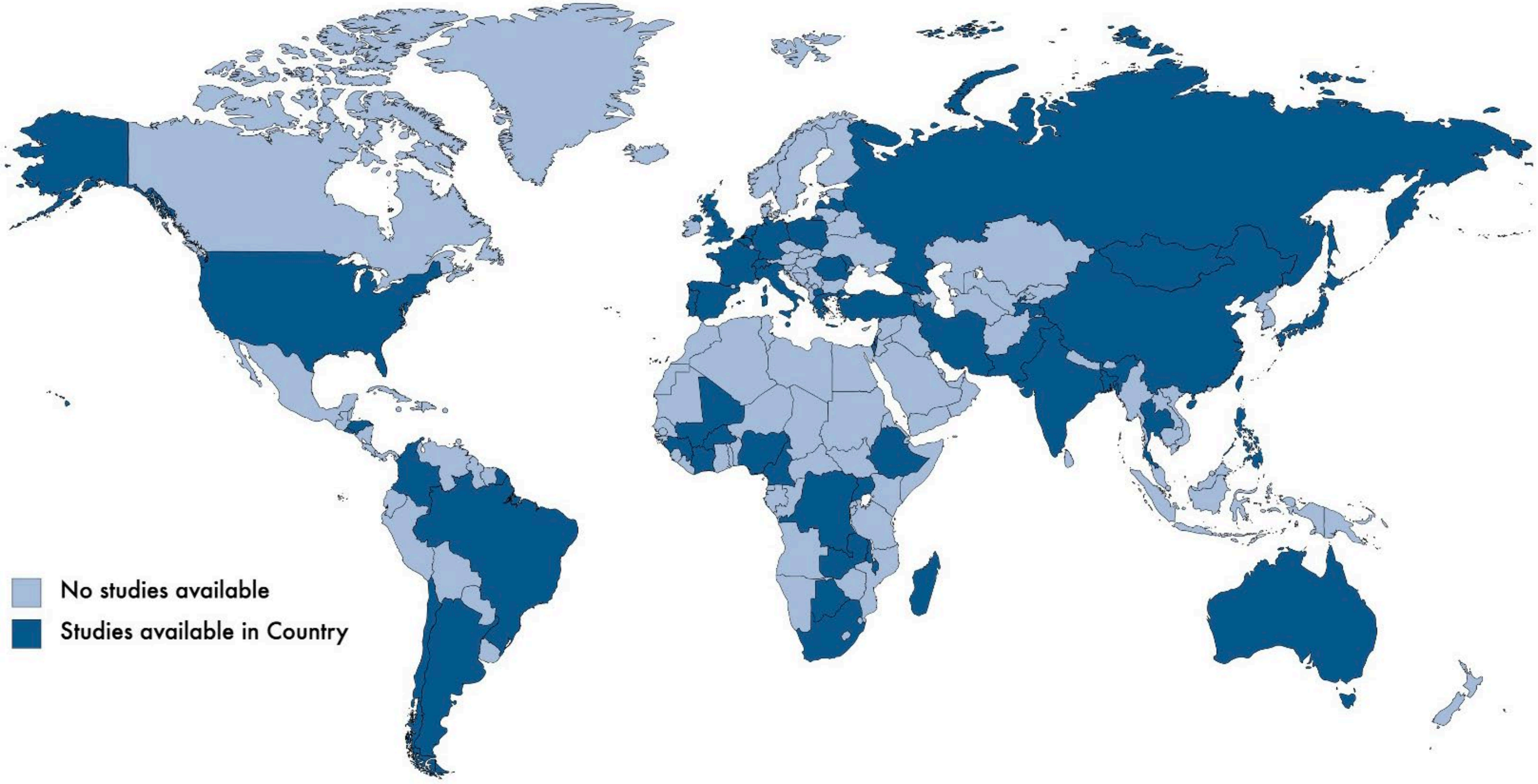

14. Supplementary Figure 2. Tuberculosis incidence per 100 thousand person-years among prisoners in different detention facilities in countries with cohort-level data.

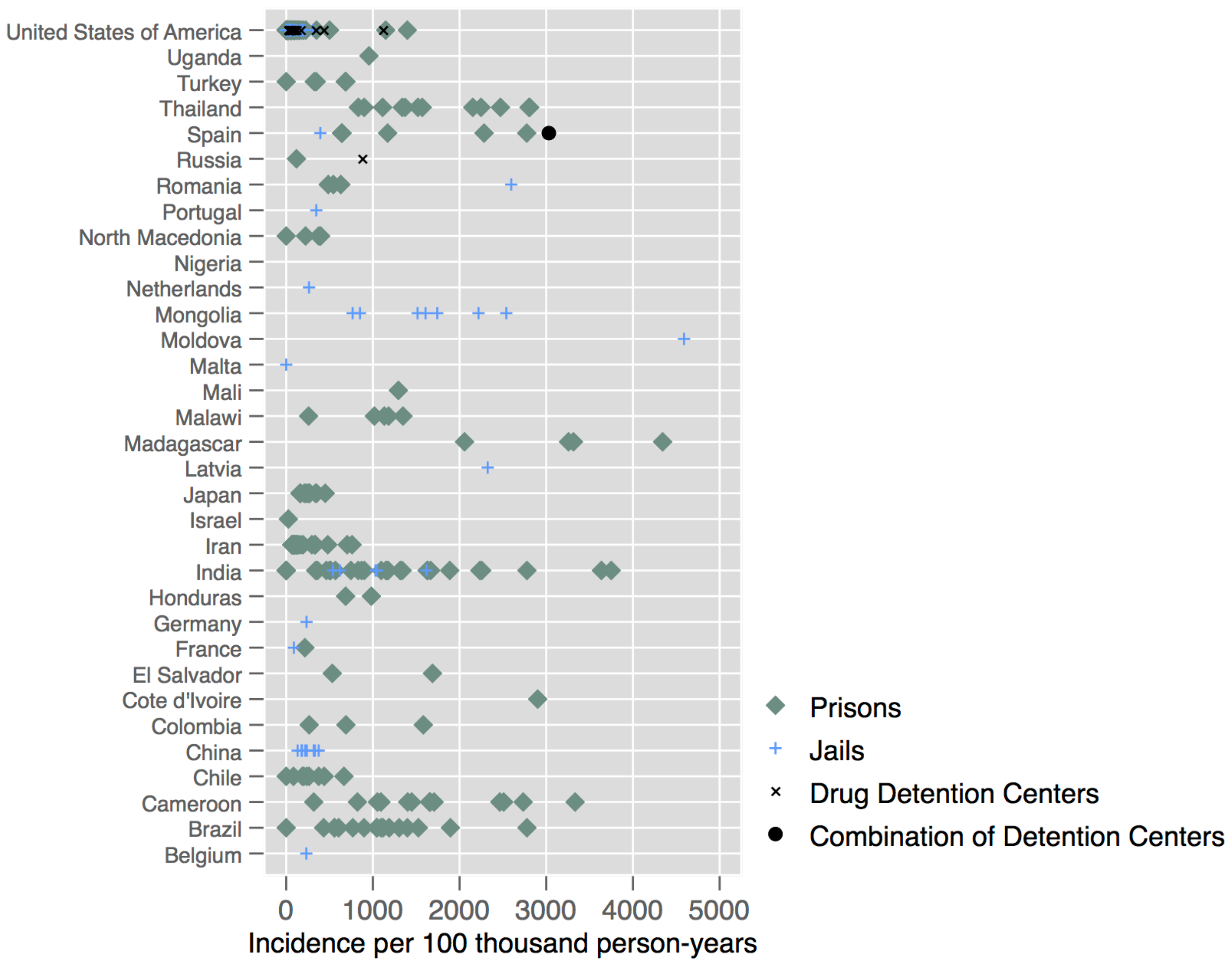

15. Supplementary Figure 3. The relationship between with study-specific tuberculosis incidence in prisons and tuberculosis incidence in the general population.

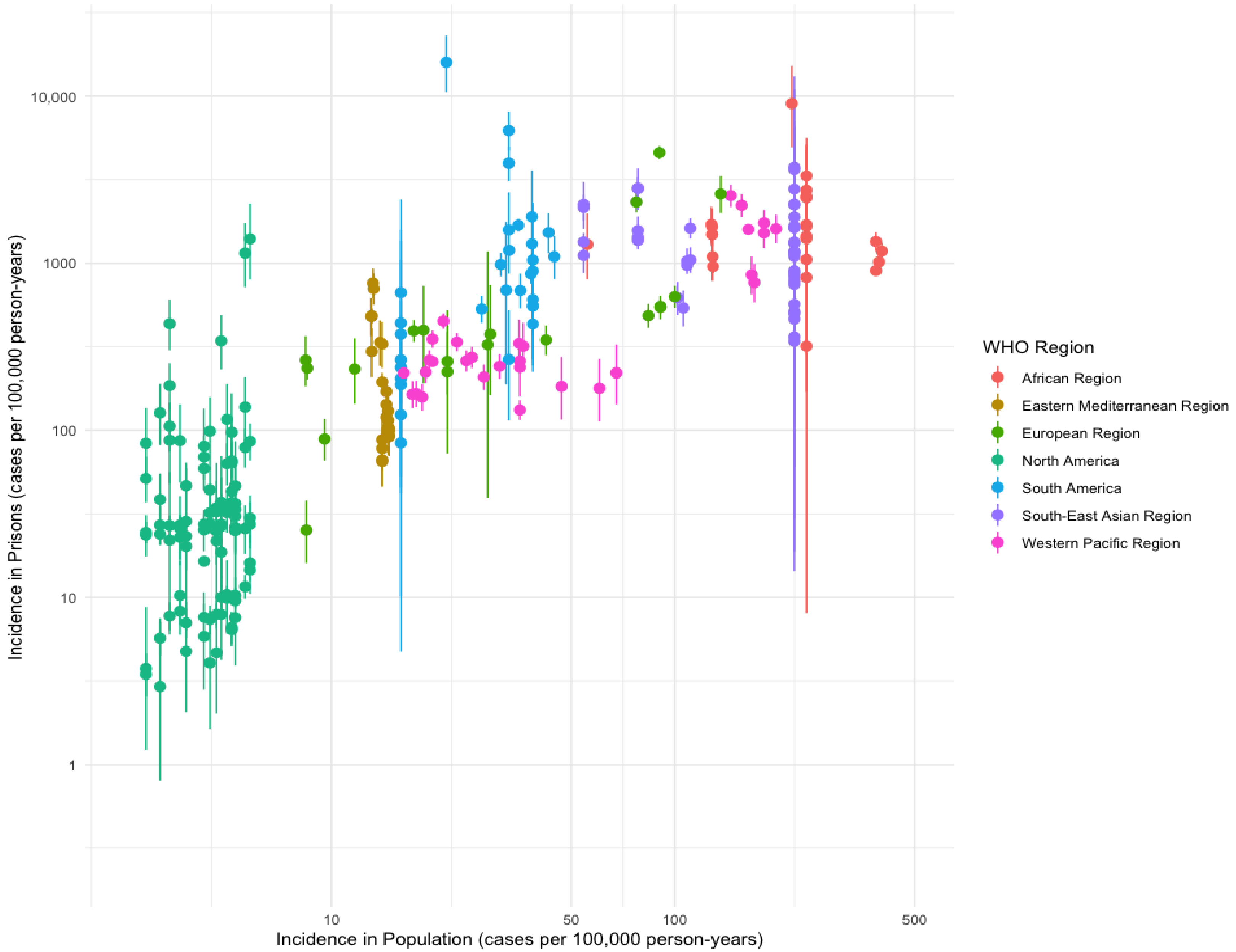

Abbreviations: WHO, World Health Organization.

Each dot represents a distinct sub-study level incidence rate per 100,000 person-years. Only studies measuring tuberculosis incidence were included in this graph. Both x- and y-axes are on a log scale. Tuberculosis incidence at the population-level was taken from World Health Organization estimates of country-level incidence. Population-level estimates were taken from World Health Organization estimates of country-level markers. For case-notification studies, we used case notification rates at the country-level reported to the World Health Organization by country officials; for non-case notification incidence studies, we used incidence rates at the country-level. We were only able to studies at or after 2000 because the World Health Organization does not provide incidence estimates for some countries before this time. The World Health Organization classifies the Americas as one region – due to substantial differences in tuberculosis burden among incarcerated populations in North and South America, we separated out this region.

16. Supplementary Table 6. Prevalence and increased odds of tuberculosis among persons living with HIV deprived of liberty

First Author	Implementation Year	Country	Persons living with HIV			Persons not living with HIV		
			Number, prevalent cases	Number, prisoners	Prevalence, 100,000 persons	Number, prevalent cases	Number, prisoners	Prevalence, 100,000 persons
Morasert	2015	Thailand	7	61	11,475.41	70	753	9,296.15
Tsegaye Sahle	2014-2016	Ethiopia	3	433	692.84	12	10374	115.67
Salazar-De La Cuba	2016	Peru	36	250	14,400.00	1718	69640	2,466.97
Pelissari	2014-2016	Brazil	18	278	6,474.82	107	4665	2,293.68
Jordan	2015	South Africa	63	2113	2,981.54	46	5454	843.42
Martin	1989-1990	Spain	8	252	3,174.60	2	84	2,380.95
Carbonara	2000-2001	Italy	1	27	3,703.70	1	311	321.54
Sanchez	2002	Brazil	6	21	28,571.43	35	935	3,743.32
Moges	2011	Ethiopia	9	19	47,368.42	17	231	7,359.31
Noeske	2003-2004	Cameroon	4	111	3,603.60	18	956	1,882.85
Farhoudi	2013-2014	Iran	5	85	5,882.35	…	…	…
Winetsky	NA	Tajikistan	4	12	33,333.33	55	1301	4,227.52
Al-Darraji	2013-2015	Malaysia	39	659	5,918.06	…	…	…
Sanchez	1991-1992	Spain	8	157	5,095.54	4	687	582.24
Telisinghe	2009-2010	South Africa	15	224	6,696.43	19	682	2,785.92
Al-Darraji	2012-2013	Malaysia	15	127	11,811.02	19	315	6,031.75

Seri	2015	Ethiopia	1	16	6,250.00	16	741	2,159.24

	$N_{studies}$	$N_{countries}$	Pooled TB prevalence, persons living with HIV (95% CrI)	Pooled Odds Ratio; persons living and not living with HIV (95% CrI)
Pooled values	17	11	8210 (3800–15210)	3·6 (2·0–5·9)

17. Supplementary Table 7. Incidence and increased risk of tuberculosis among persons living with HIV deprived of liberty.

			Persons living with HIV			Persons not living with HIV		
**First Author**	**Implementation Year**	**Country**	**Number, incident cases**	**Total person-years of follow-up**	**Incidence per 100 thousand person-years**	**Number, incident cases**	**Total person-years of follow-up**	**Incidence per 100 thousand person-years**
Chigbu	2006	Nigeria	14	83	16,867.5	0	72	0
Baillargeon	1999-2000	United States	10	2,533	394.8	29	147,151	19.7
Martin	1991-1999	Spain	16	420	3,809.5	7	3,118	224.5
Hernandez-Leon	2010-2011	Mexico	28	176	15,936.3	…	…	…
Ferreira	1992-1993	Brazil	16	161	9,937.9	4	559	715.6
Solera	1990-1992	Spain	4	58	6,932.4	0	75	0
